# Autophagy, NET formation, and inflammation crosstalk in thrombotic autoimmune diseases

**DOI:** 10.3389/fimmu.2026.1844170

**Published:** 2026-05-25

**Authors:** Tanja Muralt, Sara M. Buonomo, Mario P. Tschan, Monica Schaller

**Affiliations:** 1 Department of Hematology and Central Hematology Laboratory, Inselspital, Bern University Hospital, University of Bern, Bern, Switzerland; 2 Department for BioMedical Research (DBMR), University of Bern, Bern, Switzerland; 3 Graduate School for Cellular and Biomedical Sciences, University of Bern, Bern, Switzerland; 4 Experimental Pathology, Institute for Tissue Medicine and Pathology (ITMP), University of Bern, Bern, Switzerland

**Keywords:** autophagy, immune response, inflammation, NETs, thrombotic autoimmune diseases

## Abstract

Neutrophil extracellular traps (NETs), released by activated neutrophils, are essential components of innate immunity, capturing and neutralizing pathogens. Dysregulated NET formation can both initiate and perpetuate inflammation, autoimmunity, and thrombosis. When NETs are excessively produced or insufficiently cleared, they expose autoantigens, fuel cytokine and interferon responses, and induce endothelial damage. In addition, NETs promote platelet adhesion, activate coagulation, and impair fibrinolysis, thereby establishing them as central drivers of immune-mediated thrombotic pathology.

This review focuses on three representative thrombotic autoimmune diseases, namely immune thrombocytopenia (ITP), heparin-induced thrombocytopenia (HIT), and immune-mediated thrombotic thrombocytopenic purpura (iTTP), in which pathogenic autoantibodies and persistent NETs contribute to both thrombocytopenia and thrombosis. In ITP, endothelial activation and excessive NET release are associated with increased thrombotic risk. In HIT, platelet factor 4 (PF4)-NET complexes enhance thrombus formation while showing resistance to DNase-mediated degradation. Lastly, in iTTP, elevated levels of NET components correlate with platelet consumption and disease severity. Autophagy emerges as a central regulatory mechanism that shapes NET formation and immune activation. While autophagy supports pathogen clearance and maintains immune homeostasis, its dysregulation can amplify NET formation, sustain chronic inflammation, and promote loss of tolerance. This review proposes an interconnected model in which autophagy, NETs, and inflammation mutually reinforce one another, thereby driving thrombotic autoimmune diseases. Furthermore, it explores pharmacological agents targeting autophagy as potential treatment approaches aimed at disrupting the autophagy-NET-inflammation crosstalk and restoring immune homeostasis.

## 1 Introduction

Neutrophil extracellular traps (NETs), released by activated neutrophils, consist of decondensed chromatin fibers and antimicrobial proteins derived from neutrophil granules. As important effector mechanisms of the innate immune response, NETs entrap and help eliminate invading microorganisms (1). Various stimuli, including bacteria, fungi, viruses, parasites, and activated platelets, can induce NET formation thereby helping to prevent pathogen dissemination (2–4). Endogenous triggers of NET formation include damage-associated molecular patterns (DAMPs), immune complexes, pro-inflammatory cytokines, complement proteins, and chromatin modifications, specifically histone deamination or citrullination via peptidylarginine deiminase 4 (PAD4). NET formation must be tightly regulated to prevent pathological consequences, as dysregulated NET release can promote infections or sepsis, resulting in tissue damage and subsequent killing of epithelial and endothelial cells (5–8). Moreover, NETs contribute to coagulation abnormalities by promoting thrombosis, which may lead to microvascular occlusion. An imbalance between NET production and degradation, leading to NET accumulation, has been shown to stimulate the release of proinflammatory cytokines from endothelial cells, monocytes, macrophages, and dendritic cells (3, 9). This process further amplifies inflammation, tissue injury, and immune disorders.

As reviewed by Darrah and Andrade (10), distinct NET components externalized during NET formation act as key autoantigens, evoking autoantibody responses through the activation of B and T cells, thus promoting the development of autoimmune diseases. Autoimmunity has been linked to NET formation associated with plasma membrane rupture, which is triggered by receptors such as Toll-like receptors (TLRs), Fc-receptors that bind antibodies, and complement receptors. Upon activation, calcium released from the endoplasmic reticulum contributes to NADPH oxidase-dependent reactive oxygen species (ROS) production, thereby initiating NET formation. In animal models, activated plasmacytoid dendritic cells (pDCs) associated with NETs have been shown to enhance type I interferon (IFN-I) expression, thereby driving autoimmune pathology and exacerbating renal damage induced by neutrophil-specific autoantibodies (3). In addition, NETs directly interact with and prime CD4^+^ T cells by lowering their activation threshold, which enhances subsequent antigen specific T cell responses and enables T cell activation in response to otherwise suboptimal stimuli (11). These findings demonstrate that key components of the immune system, including pDCs and T cells, interact with NETs in a manner that may exacerbate the autoimmune condition.

Mounting evidence suggests that autophagy represents a third key process involved in autoimmunity. Autophagy is a highly conserved cellular stress response mechanism that mediates the degradation and recycling of proteins, macromolecules, and organelles to support cellular fitness (12). The outcome of autophagy ranges from modulating cell-intrinsic processes to regulate cell type-specific innate and adaptive inflammatory signaling pathways. It facilitates intercellular communication by promoting the secretion of soluble mediators, thereby contributing to the disease progression of immune-related disorders, such as autoimmune diseases, neurodegenerative conditions, inflammatory bowel disease, and metabolic disorders (12–14). Furthermore, autophagy plays a crucial role in host defense by aiding in pathogen clearance, regulating inflammatory responses, maintaining lymphocyte homeostasis, and enabling antigen presentation (12, 13). These key processes collectively influence immune regulation and disease development.

This review highlights current evidence supporting the involvement of three key mechanisms that regulate immune responses - NETs, autophagy, and inflammation - in the pathogenesis of thrombotic autoimmune diseases ITP, HIT, and iTTP. A plausible model proposes that infection-induced NET formation promotes vascular injury and, in combination with a dysregulated autophagy, amplifies the autoimmune responses. Circulating NETs further enhance coagulation, thrombosis, and vascular occlusion, which are hallmark features of the thrombotic autoimmune diseases discussed here (15–18).

**Figure 1:**
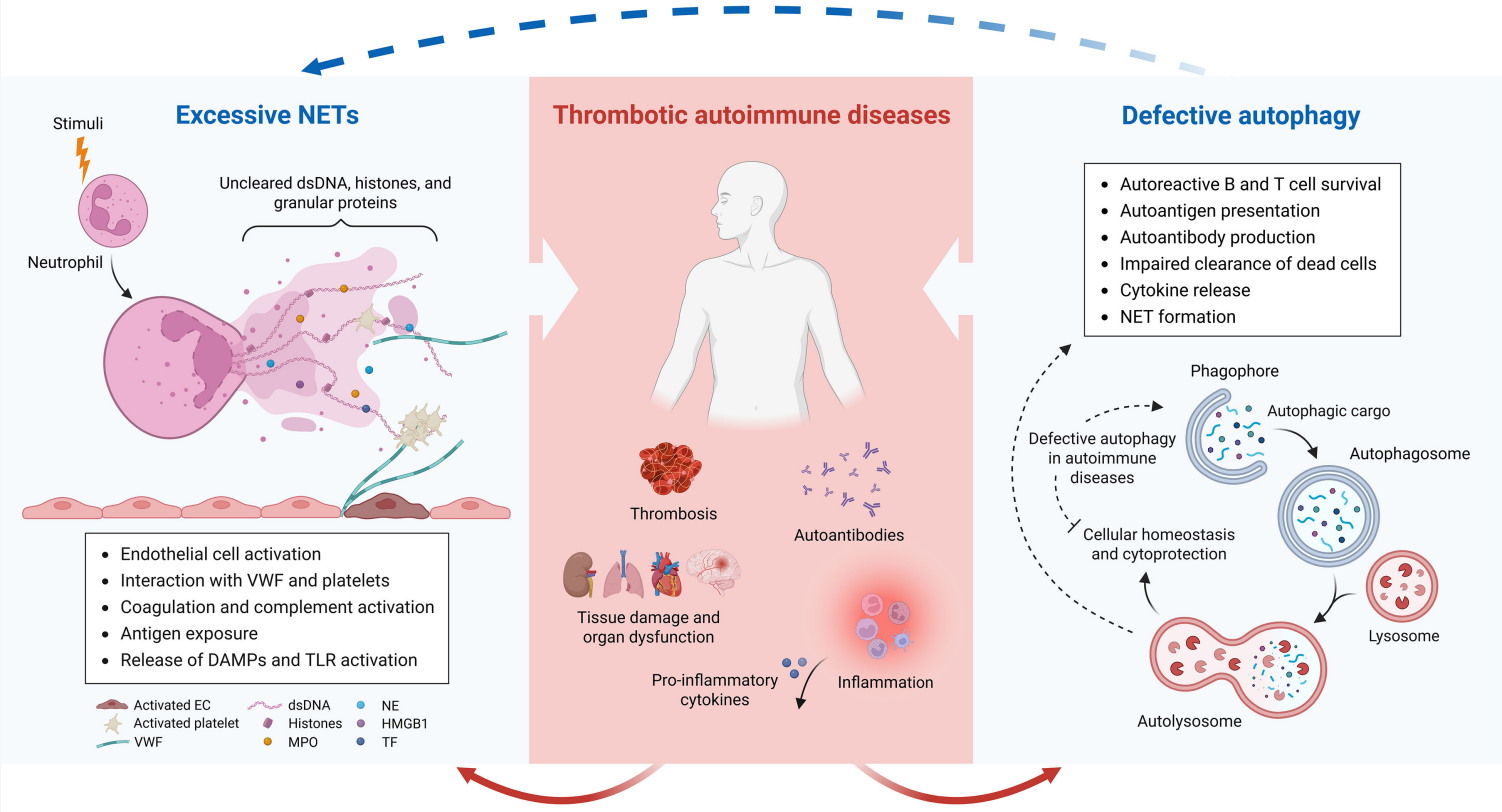
Crosstalk of NETs, autophagy, and inflammation driving thrombotic autoimmune diseases. Stimuli that activate neutrophils include pathogens, DAMPs, pro-inflammatory cytokines, immune complexes, activated platelets, and complement protein C5a. NETs, neutrophil extracellular traps; DAMPs, damage-associated molecular patterns; dsDNA, double-stranded DNA; MPO, myeloperoxidase; NE, neutrophil elastase; HMGB1, high mobility group box 1; TF, tissue factor; EC, endothelial cell; VWF, von Willebrand factor; DAMPs, damage-associated molecular patterns; TLR, Toll-like receptors. Created with BioRender.com.

While the contribution of NETs and inflammatory pathways to these disorders is increasingly recognized, the role of autophagy remains insufficiently explored. We propose that autophagy is a commonly dysregulated mechanism in these three thrombotic autoimmune disorders and hypothesize that normalization of autophagy may attenuate NET formation, autoantigen presentation, autoantibody production, endothelial release of prothrombotic mediators such as ultra-large von Willebrand factor (VWF), and platelet-rich thrombus formation (**Figure 1**).

This review focuses on ITP, HIT, and iTTP as they represent a mechanistically complementary spectrum of autoimmune thrombotic disorders. Collectively, these diseases reflect a convergence of immune dysregulation, platelet dysfunction, and thromboinflammation, providing a focused framework to examine the roles of autophagy and NETs. In this context, NETs are primarily implicated in thrombosis in HIT and iTTP, whereas autophagy appears to act across all three disorders through regulation of platelet production, immune signaling, and NET formation (**Table 1**). We therefore aimed to determine whether these immunothrombotic pathways, involving NETs and autophagy could be targeted therapeutically, as it has been explored in other broader systemic thromboinflammatory conditions, such as sepsis or systemic autoimmune diseases including systemic lupus erythematosus (SLE).

**Table 1:** ITP, HIT, and iTTP as a spectrum of immunothrombosis: comparative roles and strength of evidence for NETs and autophagy.

Features	ITP	HIT	iTTP
Autoantigen	GPIIb/IIIa	PF4-heparin complexes	ADAMTS13
Primary phenotype	Bleeding - thrombosis	Thrombosis - bleeding	Microvascular thrombosis
Strength of evidence for NET involvement	Moderate (inflammatory contribution, less central)	Strong (platelet-neutrophil activation, immunothrombosis)	Emerging (intravascular NET deposition in microthrombosis)
Strength of evidence for autophagy involvement	Moderate (megakaryocyte and platelet survival)	Limited (platelet activation, immune signaling)	Limited-emerging (endothelial/immune stress context)
Primary prothrombotic mechanism	Secondary (inflammation-driven contribution to thrombosis)	FcγRIIa-mediated neutrophil and platelet activation, thrombin generation	UL-VWF-mediated platelet aggregation (ADAMTS13 deficiency)

ITP, immune thrombocytopenia; HIT, heparin-induced thrombocytopenia; iTTP, immune-mediated thrombotic thrombocytopenic purpura; NETs, neutrophil extracellular traps; PF4, platelet factor 4; GPIIb/IIIa, glycoprotein IIb/IIIa; ADAMTS13, a disintegrin and metalloprotease with thrombospondin type 1 motif, member 13; FcγRIIa, Fc gamma receptor II alpha; UL-VWF, ultra-large von Willebrand factor. The findings presented in this review indicate that NET formation is more strongly linked to thrombotic phenotypes such as HIT and iTTP, while autophagy-related mechanisms are likely operative across all three disorders, contributing to platelet production, immune regulation, and cellular stress, and thus representing potential common therapeutic targets. Strength of evidence is based on the availability of experimental translation and clinical data.

## 2 NETs - autophagy - inflammation crosstalk

### 2.1 NETs biology

The main NET component is double-stranded DNA (dsDNA) decorated with granular enzymes, including myeloperoxidase (MPO), neutrophil elastase (NE), cathepsin G, and proteinase 3 (PR3), as well as antimicrobial peptides such as defensins and LL-37, high mobility group box 1 (HMGB1), and histones, which are frequently post-translationally modified, for example by citrullination (1). Activation of endothelial cells and platelets in response to inflammatory stimuli or vessel injury triggers the release of VWF and upregulation of P-selectin, which recruit and activate neutrophils and initiate NET formation (16, 18–21). Platelet degranulation links inflammation and thrombosis by promoting immune cell recruitment and activation, while releasing VWF and fibrinogen to drive platelet aggregation (22). Neutrophils and released NET components, particularly DNA, bind directly to VWF, anchoring them to the vessel wall and creating a scaffold that further promotes platelet activation and adhesion, as well as fibrin deposition, highlighting their prothrombotic role (16, 18–21, 23). In addition, several NET components directly or indirectly modulate the coagulation cascade. MPO and neutrophil serine proteases, including NE and cathepsin G, can inactivate anticoagulant proteins such as tissue factor pathway inhibitor (TFPI) and thrombomodulin, thereby promoting coagulation through extrinsic and, indirectly, intrinsic pathway activation (15, 17). Histones activate platelets via TLR2/4 and induce platelet-dependent thrombin generation (24). In animal models, NETs were shown to disrupt blood flow, damage the endothelium, and promote platelet aggregation (25). Consistent with this, thrombus formation and vascular occlusion was prevented by DNase treatment. NET containing thrombi are resistant to conventional fibrinolysis and require both tissue plasminogen activator (tPA)-mediated fibrin degradation and DNase I-mediated digestion of extracellular DNA for efficient dissolution. Moreover, NETs provide a surface for complement activation, whereas complement components, such as C5a, in turn stimulate neutrophils to release additional NETs (17, 26). Clearance of NETs is less well understood. Impaired NET degradation has been shown to drive inflammation and thereby cause autoimmunity. In SLE, plasma-derived DNase I predominantly degrades NET-associated DNA, whereas NET proteins continue to persist much longer, indicating that other clearance mechanisms are involved (27). Macrophages are thought to play a key role in NET protein removal (28). Despite the removal by phagocytes, NET components, especially NE and histones, can persist for several months on the endothelium of the microcirculation (29). Persisting histones may potentially trigger a secondary wave of NET formation and interleukin (IL)-1β release, which is linked to increased endothelial activation and platelet accumulation (29). This phenomenon has been demonstrated *in vivo* in liver sinusoids following endotoxemia and is proposed to amplify NET- and IL-1β-mediated responses to subsequent infections (29).

Several NET components, including DNA, histones, and HMGB1, function as DAMPs that are recognized by pattern recognition receptors such as TLR and NOD-like receptors (NLR), leading to the activation of inflammatory signaling pathways (30). Due to their proteolytic and oxidative functions, NET components have direct cytotoxic effects on host cells. In a murine sepsis model, histones were highly cytotoxic to vascular endothelial cells, promoting inflammatory responses and directly contributing to lethal outcomes (31). Similarly, Saffarzadeh et al. (32) demonstrated that NET-induced cytotoxicity in epithelial and endothelial cells is mediated by histones and MPO, contributing to lipopolysaccharide (LPS)-induced lung injury in mice. When bound to DNA, NE is protected from plasma-mediated neutralization, thereby preserving its proteolytic activity and capacity to induce cell necrosis, as observed in liver vasculature of mice following intravascular infection with methicillin-resistant *Staphylococcus aureus* (21). Administration of DNase I degraded NET-associated DNA but only partially removed histones and NE from the liver sinusoids, where these components remained directly associated with VWF (21). Consistently, blockade of VWF or administration of a disintegrin and metalloprotease with thrombospondin type 1 motif, member 13 (ADAMTS13), responsible for cleaving VWF, markedly reduced NE and histone levels, as well as DNA deposition, and consequently diminished tissue necrosis (16, 21). Thus, DNase I alone was clearly less effective at removing NET components and mitigating liver injury than either inhibition of VWF or complete genetic deletion of NE or PAD4, key enzymes involved in NET formation. Collectively, these findings indicate that NETs, and in particular NE, are responsible for most tissue injury in this model, rather than the pathogen itself.

### 2.2 NETs in autoimmunity

The breakdown of immune tolerance to self-antigens is a fundamental event in the development of autoimmunity. NETs contribute to this process by exposing autoantigens that drive autoantibody production, including antibodies targeting citrullinated proteins in rheumatoid arthritis (RA), dsDNA in SLE, and MPO and PR3 in anti-neutrophil cytoplasmic antibody (ANCA) associated vasculitis (AAV) (10, 33). Notably, the role of NETs in SLE has been extensively investigated. Impaired NET degradation due to reduced DNase I activity prolongs NET persistence and contributes to disease pathogenesis (27, 33). Furthermore, splenic neutrophils have been shown to promote immunoglobulin class switching and somatic hypermutation in B cells, thereby supporting antibody diversification and production (34). Similarly, in patients with AAV, reduced DNase I activity impairs NET degradation, resulting in persistent NETs that promote ANCA production (35). In turn, circulating neutrophils can be activated by autoantibodies such as ANCAs, which causes excessive NET formation and promotes antigen presentation by dendritic cells (DCs) to T cells, B cell activation and survival, and thereby a continuous antibody production (35). NETs not only prime T cells (11) but also directly induce TLR2-dependent T cell differentiation into the proinflammatory T helper cells 17 (Th17) subset, known to be important for neutrophil recruitment and strongly associated with several autoimmune diseases (35, 36). In SLE, NET components have been shown to activate pDCs. Elevated levels of NETs decorated with the alarmin IL-33, which is proteolytically cleaved by NE into its bioactive form, have been detected in inflamed skin and kidney tissue biopsies, as well as in serum samples from patients with active SLE (37). These levels significantly correlated with disease activity. IL-33 containing NETs released by neutrophils from SLE patients that were cultured with SLE-derived immune complexes, strongly induced IFN-α production in pDCs via the IL-33 receptor ST2L and regulatory factor 7. In line with these findings, neutrophils of pediatric SLE patients stimulated with anti-ribonucleoprotein (RNP) antibodies purified from SLE serum released NETs containing LL37 and HMGB1, which induced TLR9-dependent IFN-α production in pDCs (38). These neutrophils were primed to induce NET formation, as evidenced by their IFN-I and TLR-signaling transcriptional phenotype, compared to healthy neutrophils, which failed to release NETs. Thus, NET components contribute to the IFN-I-driven inflammatory milieu present in SLE and demonstrate that autoantibodies play a key role in the activation of neutrophils and NET release. Furthermore, NETs activate macrophages via the NLRP3 inflammasome, leading to the release of IL-1β and IL-18 (39). Despite these insights, the shared mechanisms underlying NET-driven autoimmune responses need further investigation. Findings from various *in vitro* and *in vivo* studies support the concept of a vicious cycle of inflammation and autoimmunity (25, 40). Taken together, NET components generated during inflammation may serve as autoantigen sources, initiating dysregulated immune responses marked by autoantibody production. These autoantibodies, along with immune complexes, could in turn promote additional NET formation, fueling a self-perpetuating loop of autoimmune inflammation (33, 40). On top, the dysregulated pro-inflammatory cytokine milieu present in many autoimmune and pathological conditions further exacerbates neutrophil migration, activation, and NET formation (4) (**Figure 1**, left panel).

### 2.3 NETs in thrombotic autoimmune diseases

Prevention of hematologic autoantibody-mediated thrombotic disorders, ITP, HIT, and iTTP, remains challenging and is managed by nonspecific immunosuppressive therapies aimed at blocking antibody generation and inflammation, with variable outcomes for most patients (41–43). Common to all three disorders is the initial concurrence of autoantibodies and their respective self-antigenic targets in the absence of thrombotic events. This suggests that conformational changes in the relevant autoantigens, namely platelets in ITP, PF4/heparin complexes in HIT, and the transition of ADAMTS13 from its closed to open form in iTTP, are required for recognition by pathogenic antibodies, leading to thrombocytopenia and severe thrombosis characteristic of all three autoimmune conditions. Although autoimmune responses are known to play a partial role in the thrombotic events associated with ITP, HIT, and iTTP, the precise mechanisms remain unclear. Given the established role of NETs in thrombosis and the frequent occurrence of infections preceding disease onset of ITP, HIT, and iTTP, this review aims to elucidate how NETs specifically contribute to the prothrombotic state reported in these autoimmune conditions, which constitute the central focus of this review.

In ITP, thrombocytopenia is primarily caused by autoantibodies directed against various platelet and megakaryocyte surface glycoproteins (GPs), most commonly GPIIb/IIIa, in combination with T cell-mediated platelet destruction and cytokine imbalances promoting the autoimmune state (43). Beyond bleeding events, patients with ITP also display a paradoxical thrombotic risk. Increased levels of endothelial activation markers, including ICAM-1, together with enhanced NET formation detected in plasma of ITP patients, support a role for NET-associated endothelial and neutrophil activation in the prothrombotic state of ITP (44, 45). Activated neutrophils may further amplify disease progression through increased B cell activating factor (BAFF) production and enhanced B cell responses (46), alongside dysregulated Th17 responses and inflammasome activation, which together promote inflammation and thrombosis (47). However, direct evidence defining the specific role and underlying mechanisms of NET formation in ITP remains limited; this gap highlights the need for further studies to clarify their contribution to disease pathogenesis.

HIT is the most common drug-induced immune-mediated thrombocytopenia, occurring in ∼1% of patients, typically 3–6 days after treatment with unfractionated heparin (UFH) (41, 48–50). Autoantibodies are targeted against PF4 in a complex with heparin, or other polysaccharides and cellular glycosaminoglycans (GAGs). Autoantibodies binding to the PF4/heparin complexes in the circulation crosslink FcγRIIa receptor on the surface of platelets, promoting platelet activation and aggregation and clot formation responsible for the observed platelet count drop (50). Binding of these immune complexes to FcγRIIa activates both platelets and neutrophils. In neutrophils, FcγRIIa activation promotes ROS generation, neutrophil adhesion, and NET release, while platelet P-selectin further supports the formation of neutrophil-platelet aggregates (50, 51). Released NETs provide a scaffold for platelets and coagulation factors and appear to be mechanistically required for thrombus formation in HIT models (50, 51). Gollomp et al. (50) elegantly showed, using a microfluidic system, that PF4 can bind directly to NETs, forming compact complexes that are resistant to DNase degradation and, thus, persist in the circulation, continuously exposing autoantigens. In addition, PF4-NET complexes can selectively bind HIT antibodies, which further protects them from DNase I digestion. Elevated levels of NET markers, including citrullinated histone H3 (citH3), MPO, elastase and cell free DNA, were found in plasma of HIT patients, supporting the important contribution of NETs to the prothrombotic state in HIT (50, 51). NET formation induced by HIT immune complexes and subsequent thrombus formation was later described as a NADPH oxidase- and ROS-dependent mechanism, associated with mitochondrial dysfunction (52). Similar FcγRIIa-dependent platelet-neutrophil interactions and NET-driven thrombotic mechanisms have been described in vaccine-induced immune thrombotic thrombocytopenia (VITT), a rare PF4-centered thrombotic autoimmune disorder that shares key mechanistic features with HIT (53–55). VITT occurs predominantly after vaccination with adenoviral vector-based vaccines such as ChAdOx1-S (AstraZeneca) and Ad26.COV2.S (Johnson & Johnson/Janssen), and in rare cases following natural adenovirus infection (56, 57). It is mediated by highly stereotyped anti-PF4 antibodies that form immune complexes with tetrameric PF4, arising from a misdirected secondary immune response against adenoviral protein pVII in genetically predisposed individuals. These PF4-immune complexes activate platelets and neutrophils via FcγRIIa crosslinking, promoting robust NET formation (53, 54). NETs, in turn, contribute to thrombus propagation by enhancing neutrophil-endothelial interactions and providing a DNA scaffold that binds PF4, thereby facilitating PF4 immune complex formation and amplifying immunothrombosis, in a manner analogous to HIT (53). This process has been shown to mediate thrombus formation *in vitro* in a microfluidic system, as well as driving thrombosis *in vivo* (54). Consistent with these findings, NET markers were significantly elevated in serum and plasma of VITT patients (53, 54). Carnevale et al. (55) also reported increased procoagulant markers, such as soluble tissue factor (TF), D-dimer, and prothrombin fragments. In addition, expression of PAD4, citH3, TF, and cathepsin G were markedly increased in a thrombus of a VITT patient, indicating PAD4-dependent NET formation (55). Greinacher et al. (53) further observed abundant activated neutrophils and NET structures in cerebral sinus vein thrombi of VITT patients. *In vitro* studies confirmed increased NET formation, TF activation, thrombin generation, and thrombus growth in the presence of plasma from VITT patients, highlighting the cooperative interaction between platelets and neutrophils (55). Importantly, blocking FcγRIIa inhibited NET formation, platelet aggregation, and thrombus formation *in vitro* and *in vivo* (53–55). Collectively, these observations suggest that FcγRIIa-dependent platelet-neutrophil interactions and NET formation play a key role in the development of thrombosis in VITT, as observed in HIT.

Immune-mediated TTP is characterized by autoantibodies against ADAMTS13, resulting in severe ADAMTS13 deficiency and accumulation of ultra-large VWF multimers, the hallmark of acute iTTP (42). In this context, Fuchs et al. (58) reported that histone/DNA complexes, MPO, and the proinflammatory mediator S100A8/A9 released from neutrophils were significantly elevated in plasma during acute disease, possibly contributing to key clinical features such as thrombocytopenia, microvascular thrombosis, organ damage, and potentially mortality (58). In line with these findings, Sui et al. (59) reported that elevated levels of these markers in iTTP patients at admission are associated with a more severe disease course, increased risk of mortality and disease recurrence after clinical remission. Increased NET markers in plasma from iTTP patients in the acute phase were linked to an increased percentage of NET producing neutrophils, accompanied by a reduced potential of inducible NET formation, which could be restored after therapy (60). Evidence that degradation of NETs by DNase is a prerequisite to prevent thrombosis, stems from *Dnase1^–/–^ Dnase1l3^–/–^* mice, which displayed features of thrombotic microangiopathies (TMAs) and intravascular coagulation, including schistocytes, hemolytic anemia, and organ failure due to vascular occlusion (61). Consistently, NETs generated *in vitro* remained intact after exposure to patient plasma collected in the acute disease state, whereas plasma supplemented with recombinant DNase I restored NET degradation, suggesting reduced DNase I activity in acute disease (62). Additionally, Yada et al. (60) demonstrated that whole blood from patients in remission with normalized platelet counts exhibited increased thrombus formation and NET accumulation when perfused over a collagen surface. Notably, both were completely abolished in the presence of DNase I. Consistent with plasma findings, NET accumulation and thrombus formation were absent in whole blood from acute-phase patients with low platelet counts, underscoring the requirement for platelets and VWF. Beyond serving as a scaffold for platelet adhesion by retaining NET components on the damaged endothelium, VWF also interacts with neutrophil-derived enzymes. These enzymes may alter ADAMTS13 activity, either directly or indirectly, through proteolytic cleavage, chemical modification, or competitive binding to VWF (16). Such interference can lead to an accumulation of ultra-large VWF multimers, further amplifying inflammation and promoting thrombus progression, thereby emphasizing the critical role of ADAMTS13 in maintaining hemostatic balance (16).

NET formation has been implicated in antiphospholipid syndrome (APS), another thrombotic autoimmune disease characterized by antiphospholipid antibodies including anti-β2-glycoprotein I antibodies, anticardiolipin antibodies, and lupus anticoagulant (63). Immune complexes formed by these autoantibodies can induce NET formation (64). In APS patients, circulating NET markers are elevated, NET degradation is impaired, and anti-NET autoantibody levels are increased, which correlates with complement consumption and venous thrombosis (65–67).

Taken together, inflammation and NET formation contribute to the prothrombotic state observed in ITP, HIT, and iTTP by promoting neutrophil adhesion to the endothelium and subsequent migration into thrombi. Consequently, NETs constitute a unique link between inflammation, platelet aggregation, and thrombosis.

### 2.4 Autophagy-mediated NETs

Autophagy contributes to the immunological functions of neutrophils by regulating their differentiation, survival, degranulation, metabolism, and NET formation (68). Activation of autophagy has been reported to enhance spontaneous NET release (69) and to facilitate chromatin decondensation and histone citrullination (70, 71). This process is further linked to cytoskeletal dynamics, as disruption of autophagosome formation impairs NET release (70). Mitochondria-specific autophagy, termed mitophagy, is an important regulatory mechanism in neutrophils to maintain mitochondrial quality by clearing damaged mitochondria and thereby limiting mitochondrial ROS and mitochondrial DNA release, both potent inducers of NET formation (72, 73). However, autophagy-driven NET formation seems to be context-dependent: while moderate NET release may be protective, excessive NET generation can promote tissue damage and disease progression.

Evidence from other inflammatory and autoimmune conditions such as gouty arthritis, ANCA-associated vasculitis, and SLE, suggests that autophagy can regulate NET formation and influence disease severity (74–77). This is evidenced in gouty arthritis, where urate crystals have been shown to induce NET formation through an autophagy-dependent mechanism involving the key autophagy related protein 7 (ATG7) (74). In this context, autophagy modulated disease severity by facilitating resolution of inflammation. Similarly, in ANCA-associated vasculitis, ANCA antibodies were found to trigger NET formation, likely via autophagy, which facilitates NET release and may contribute to the development of autoimmunity (75, 76). In SLE, autophagy-driven NET formation has been linked to increased disease severity, possibly due to enhanced exposure of nuclear autoantigens (77). For a more in-depth overview, readers are referred to the review by Liang et al. (78), which summarizes all possible regulatory pathways of autophagy. There is evidence suggesting that NET formation may also occur independently of autophagy, as late-phase autophagy inhibitors did not impair the ability of neutrophils to release NETs (79). Importantly, direct evidence linking autophagy-mediated NET formation to ITP, HIT, or iTTP is currently lacking. Thus, the role of autophagy in these conditions remains speculative, and further studies are needed to define the specific pathways involved, with current evidence remaining largely circumstantial and limited to a presumed general modulatory role.

### 2.5 Autophagy, autoimmunity, and inflammation

Autophagy regulates several core immune functions, including inflammatory signaling, lymphocyte homeostasis, antigen presentation, and host defense, thereby influencing autoimmune pathology (13, 80, 81). In the context of lymphocyte homeostasis autophagy plays a crucial role in the survival, activation, and long-term maintenance of T and B cells (13, 80, 81). As demonstrated in mice, specific deletion of the core autophagy gene *Atg5* in mature T cells did not affect CD4^+^ T cell numbers and short-term activation (82). However, CD4^+^ T cell memory and CD4^+^ T cell-dependent humoral immune responses were substantially reduced (82). *Beclin-1* deletion increased susceptibility of CD4^+^ T cells to cell death upon TCR activation (83). These findings indicate that defects in ATG genes are responsible for immune cell death and failure to generate immune memory (82–84). Furthermore, autophagy is critical for maintaining the stability, survival, and suppressive function of regulatory T cells (Tregs), thereby supporting immune tolerance and preventing excessive immune responses. Indeed, genetic disruption of autophagy pathways (e.g., *Atg5* or *Atg7* deficiency) results in increased apoptosis, reduced *Foxp3* expression, and impaired Treg function, ultimately leading to a loss of immune tolerance (85). In addition, autophagy regulates antigen presentation through major histocompatibility complex (MHC) class II pathway in APCs including B cells and supports plasma cell differentiation and autoreactive B-cell survival (86, 87). Besides its role in pathogen clearance, which is induced by pattern recognition receptor activation and pro-inflammatory cytokines such as TNF, IL-1β, IL-6, and type I and type II IFN, autophagy also regulates pro-inflammatory signaling pathways, including the inflammasome and the secretion of inflammatory immune mediators (13, 81, 88–90). Thus, autophagy plays a regulatory role in numerous inflammatory processes that contribute to the pathogenesis of autoimmune disorders.

Autophagy comprises three major subtypes, macroautophagy, microautophagy, and chaperone-mediated autophagy (CMA), each of which may differentially contribute to immune regulation and autoimmune disease (91, 92). Macroautophagy targets damaged components in the cytoplasm through the formation of an isolation membrane (phagophore) within the cell, which engulfs intracellular cargo. This phagophore can recruit cytoplasmic components selectively or non-selectively and will later expand into a sealed, double-membrane structure, called autophagosome. This process is orchestrated with the help of two ubiquitin-like conjugation systems, ATG12 and ATG8/microtubule-associated protein 1A/1B-light chain 3 (LC3) and involves the sequential action of hierarchically organized ATG proteins that govern autophagosome initiation, formation, and maturation. The autophagosome ultimately fuses with the lysosome to form the autophagolysosome, where the sequestered substrates are degraded and recycled. Inhibitors of this pathway, such as Bafilomycin A1 and chloroquine, act by blocking the autophagolysosome formation. In contrast, microautophagy is a non-selective degradation process in which the lysosomal membrane invaginates and thereby sequesters small volumes of substrates directly into the lysosome to be degraded by proteases. CMA is a selective degradation process where specific substrates containing a consensus pentapeptide-motif (KFERQ) are recognized by chaperone proteins [heat shock protein family A member 8 (HSPA8)/heat shock cognate 70 (HSC70)] and translocated via lysosome-associated membrane protein type 2A (LAMP2A) into the lysosome (92).

Single nucleotide polymorphisms (SNP) in autophagy-related genes have been associated with several autoimmune diseases. The strongest and most consistent evidence exists for *ATG5* variants in SLE (93, 94), including lupus nephritis (95), whereas associations in RA (96, 97), Sjögren’s syndrome (98), and psoriasis (99) are less consistent. SNPs in *ATG16L1*, *IRGM*, and *ULK1* have been linked primarily to Crohn’s disease (100). Collectively, these findings highlight a connection between genetic variation in the autophagy pathway and aberrant autophagic activity in autoimmune diseases. In addition to genetic polymorphisms, dysregulated expression of ATG genes has been reported for *ATG5* in patients with SLE, RA, psoriasis, and multiple sclerosis (MS) (12, 101, 102). ATG16L1 was found to be overexpressed in DCs from psoriatic arthritis patients and seems to be involved in Crohn’s disease (12, 84). *ATG7*, together with *ATG3*, catalyzes the formation of lipidated LC3-II during autophagosome elongation and shows an increased expression profile in osteoclasts of RA patients (12). It is also associated with SLE and experimental autoimmune encephalomyelitis (EAE), an established animal model for MS (12, 103). Accumulating evidence indicates that activation of autophagy contributes to the pathogenesis of SLE and RA, with autophagic activity correlating with disease severity in both disorders (86, 104). Macroautophagy in T cells of lupus mice (MRL^lpr/lpr^ and (NZB/NZW)F1) and SLE patients was found to be dysregulated, as shown by increased numbers of autophagic vacuoles and LC3-II levels compared to controls (105). CMA and the expression levels of MHC class II molecules are highly elevated in B cells of murine lupus models and SLE patients, supporting the role of autophagy in antigen presentation, thereby promoting autoimmunity (92, 106–108). Furthermore, myeloid specific deletion of either *Atg7* or *Atg5* in mice significantly reduced the degranulation capacity of bone marrow neutrophils *in vitro* due to defective NADPH oxidase-dependent ROS production (109). Inflammation was significantly reduced in neutrophil-mediated inflammatory mouse models upon myeloid-specific *Atg7* deficiency, which also markedly attenuated the severity of autoimmune responses in EAE (109). This effect appears to result from impaired neutrophil function rather than altered antigen presentation or T cell activation. Similarly, *Atg5* deletion in myeloid cells also resulted in a reduction of the EAE phenotype (109) and loss of *Beclin-1* protected mice from developing EAE (83). However, the effects of autophagy modulation appear to be context dependent. While inhibition of autophagy has been reported to be beneficial in SLE, RA, and MS, it seems to aggravate psoriatic arthritis and Crohn’s disease (12, 110). These opposing effects may reflect differences in the cell types involved in each autoimmune disease, as well as distinct pathways that are independently up- or downregulated across various cell populations within the same individual (12, 86). To assess the role of autophagy in disease progression, severity, and its contribution to the autoimmune state it is essential to analyze immune cell types individually within their microenvironment.

It is important to stress out that studies in thrombotic autoimmune disorders remain scarce. In ITP, autophagy seems to be crucial for megakaryocyte and platelet biology and was found to be significantly reduced in platelets of affected patients (111, 112). *Beclin-1* mRNA expression was significantly increased in peripheral blood mononuclear cells (PBMCs) of newly diagnosed ITP patients with active disease compared to patients in remission and healthy controls (113). Proteomic analysis of bone marrow-derived mononuclear cells from ITP patients revealed downregulation of several ATG proteins, including HSPA8, and one upregulated protein, namely colony-stimulating factor 1 receptor (CSF1R) (114). Increased mRNA expression of the autophagy markers *LC3* and *Beclin-1*, together with reduced levels of *p62*, has been reported in whole blood samples from pediatric ITP patients and was positively correlated with the presence of antiplatelet antibodies (115). Autophagy regulates the release of VWF from Weibel-Palade bodies (WPB) of endothelial cells. Torisu et al. (116) observed that WPB were often found in close proximity to autophagosomes. *ATG7* and *ATG5* depletion in human umbilical vein endothelial cells (HUVECs) reduced basal VWF levels and led to decrease in VWF release after stimulation with histamine or vascular endothelial growth factor (VEGF) due to impaired VWF processing and maturation to WPBs. Pharmacological inhibition of autophagy with chloroquine or Bafilomycin A1 showed similar results. Consistent with these findings, endothelial-specific knockout of *Atg7* or *Atg5* in mice led to impaired epinephrine-VWF release (116). These findings indicate that autophagy in endothelial cells may be relevant in the context of iTTP (117). However, comparable studies are currently lacking for HIT and iTTP, highlighting an unmet need for further investigation.

Inflammation and autoimmunity are characterized by persistent activation of pro-inflammatory signaling pathways. Cytokines, immune complexes, and complement activation drive the activation of neutrophils, monocytes, platelets, and endothelial cells, thereby sustaining a pro-inflammatory and pro-thrombotic environment. Autophagy regulates inflammatory signaling by controlling cellular degradation, inflammasome activation, and cytokine production, while also influencing neutrophil activation and NET formation. Dysregulated autophagy may therefore sustain pro-inflammatory signaling and contribute to immunothrombosis in autoimmune diseases.

### 2.6 Potential novel treatment strategies

Given that pro-inflammatory cytokines, immune complexes, and cellular activation pathways drive thrombus formation, inflammation represents a central therapeutic target in these disorders. Accordingly, corticosteroids remain a cornerstone of treatment in ITP and iTTP. In this review, we propose autophagy as potential shared pathogenic mechanism that may be therapeutically targeted to restore immune homeostasis, although this concept requires further validation. As highlighted throughout this review, much of the current evidence is derived from studies in other autoimmune diseases, whereas data in thrombotic autoimmune conditions remain limited. Existing studies primarily focus on the contribution of inflammation and NETs to autoimmunity and thrombosis, particularly in HIT and iTTP. Evidence of autophagy dysregulation in different cell types is primarily confined to ITP. In contrast the effect of autophagy modulation on NET formation has yet to be elucidated.

**Figure 2:**
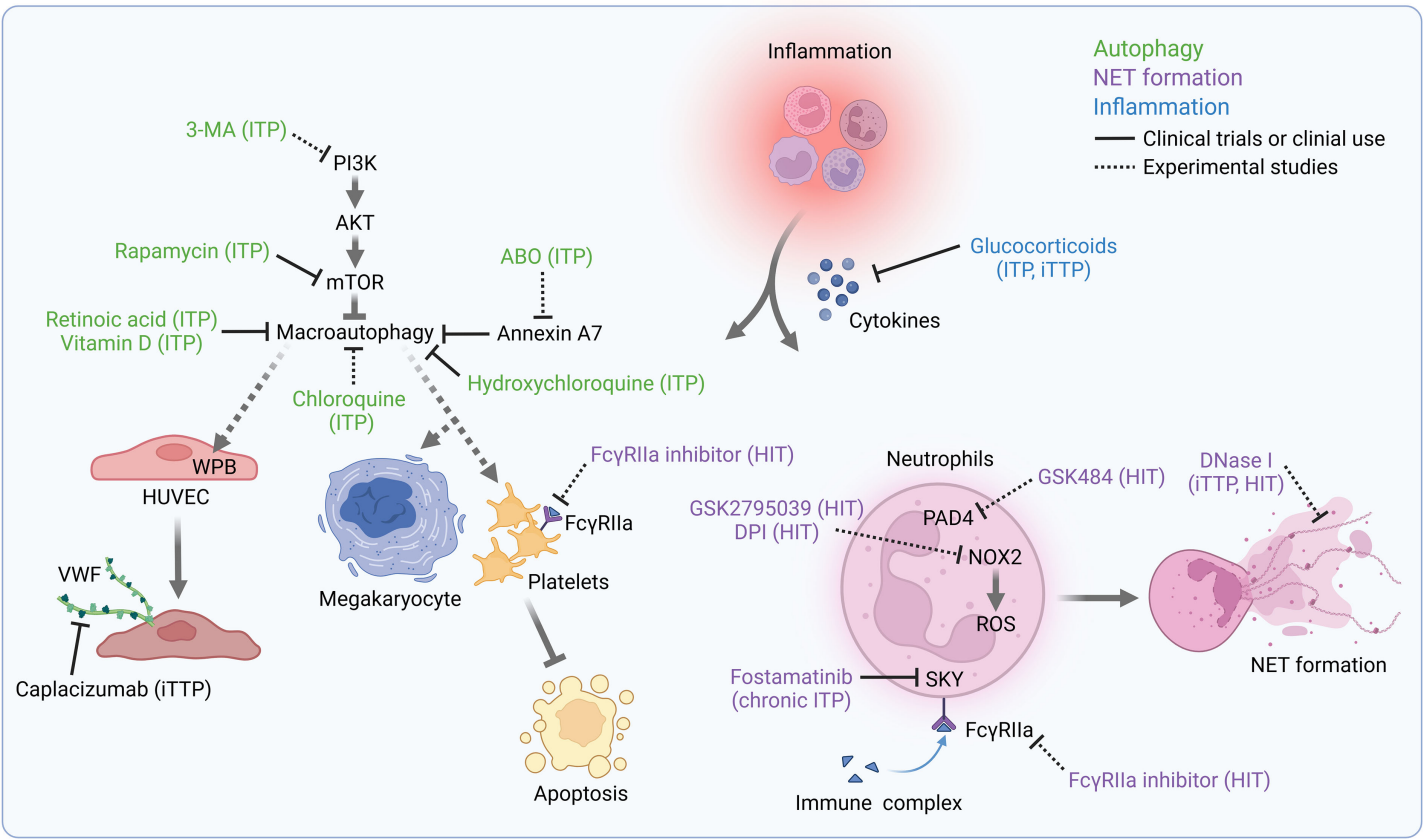
Pharmacological targeting and testable mechanistic hypotheses linking autophagy, NET formation, and inflammation in ITP, HIT, and iTTP. Current evidence for pharmacological targeting of autophagy, NET formation, and inflammation remains limited. This figure integrates available data to propose testable molecular hypotheses and summarizes therapeutic targets and agents under investigation. Evidence derived from experimental studies is indicated with dashed lines (–), including 3-MA, ABO, chloroquine, FcγRIIa inhibitor, GSK484, GSK2795039, DPI, and DNase I, whereas evidence from clinical trials (rapamycin, retinoic acid, vitamin D, hydroxychloroquine) or in clinical use (corticosteroids, fostamatinib) is represented with solid lines (—). Mechanistically, macroautophagy regulates platelet survival and function in ITP, as induction of autophagy (e.g., rapamycin, ABO) reduces platelet apoptosis, whereas autophagy inhibition (3-MA) abrogates this effect. In endothelial cells, inhibition of autophagy (e.g., chloroquine) reduces VWF processing and release, suggesting that autophagy may regulate VWF-mediated thrombus formation, a mechanism potentially relevant in iTTP. While direct evidence in ITP, HIT, and iTTP remains limited, these pathways define a testable mechanistic framework in which dysregulated autophagy in neutrophils, endothelial cells, and platelet/megakaryocyte lineages may amplify NET formation, inflammation, and thrombosis. Therapeutically, this framework highlights multiple intervention points, including inhibition of NET formation (e.g., PAD4, NOX2, DNase I), blockade of FcγRIIa/SYK signaling, modulation of oxidative stress, and targeting of autophagy pathways. The anti-VWF nanobody caplacizumab, although not directly targeting these pathways, prevents thrombus formation by blocking platelet-VWF binding and may indirectly interfere with NET-VWF interactions. This suggests that dysregulated autophagy in endothelial cells or platelets may contribute to VWF release and thrombus formation in iTTP, representing a testable hypothesis that warrants further investigation. Overall, the here depicted pathways represent a rational strategy for potential therapeutic targets to be evaluated individually or in combination. ITP, immune thrombocytopenia; HIT, heparin-induced thrombocytopenia; iTTP, immune-mediated thrombotic thrombocytopenic purpura; NETs, neutrophil extracellular traps; DNase I, deoxyribonuclease I; PAD4, peptidylarginine deiminase 4; ROS, reactive oxygen species; NOX2, nicotinamide adenine dinucleotide phosphate (NADPH) oxidase 2; DPI, diphenyleneiodonium chloride; FcγRIIa, Fc gamma receptor II alpha; SYK, spleen tyrosine kinase; HUVEC, human umbilical vein endothelial cells; WPB, Weibel-Palade bodies; VWF, von Willebrand factor; PI3K, phosphoinositide 3-kinase; AKT, serine/threonine protein kinase; mTOR, mammalian target of rapamycin; ABO, 6-amino-2,3-dihydro-3-hydroxymethyl-1,4-benzoxazine; 3-MA, 3-methyladenine.

Current evidence of drugs targeting autophagy, NET formation, and inflammation in ITP, HIT, and iTTP is limited. **Table 2** summarizes potential targets and drugs that have been investigated in the context of these diseases. The strength of evidence supporting the involvement of NETs or autophagy varies across the three diseases, ranging from strong to moderate and emerging levels (**Table 1**). A promising category includes drugs targeting FcγRIIa-mediated thrombo-inflammatory signaling. This is particularly relevant for HIT, where FcγRIIa is a key mediator of platelet and neutrophil activation by pathogenic immune complexes (**Figure 2**). Thrombi generated *in vitro* using a microfluidic system or static condition using neutrophils was blocked by FcγRIIa inhibition (51). In a HIT murine model using transgenic *FcγRIIA^+^/human PF4^+^* mice, blocking FcγRIIa completely abolished thrombus formation and significantly improved platelet count (51). Similarly, inhibition of NET formation using PAD4 inhibitor GSK484 reduced thrombosis, but did not affect thrombocytopenia, indicating that thrombosis and thrombocytopenia are two distinct processes in HIT. Consistently, PAD4-deficient *FcγRIIA^+^/human PF4^+^* mice failed to develop thrombosis when injected with HIT antibodies and heparin (50, 51). Treatment with DNase I also decreased thrombus formation but was less effective and had no effect on thrombocytopenia. Additionally, inhibition of NADPH oxidase 2 (NOX2) with DPI or GSK2795039, leading to reduced ROS production, prevented thrombus formation *in vivo* (52). The bivalent nanobody caplacizumab, which targets the VWF A1 domain, was recently approved for clinical use in iTTP (118, 119). By shielding the A1 domain, it inhibits platelet adhesion to VWF, therefore preventing the formation of VWF-platelet thrombi. Given that NET-derived DNA has been shown to interact with VWF through the A1 domain, it is plausible that caplacizumab might also reduce the prothrombotic effects of NETs (23). These findings highlight NETs as a promising new target that could improve thrombosis and clinical outcomes of HIT patients, and that FcγRIIa-dependent neutrophil activation should be investigated as well in iTTP.

**Table 2:** Current evidence on pharmacological modulation of inflammation, NET formation, and autophagy in ITP, HIT, and iTTP is summarized in this table.

Target	Drug	Disease context	References
Inflammation			
Intracellular receptor inhibitors	Glucocorticoids (Prednisone, Dexamethasone)	ITP*, iTTP*	(42, 43)
NETs			
DNA degradation	DNase I	iTTP (E), HIT (E)	(50, 51, 60)
PAD4 inhibitor	GSK484	HIT (E)	(51)
FcγRIIa inhibitor	FcγRIIa antibody	HIT (E)	(51, 52)
SYK (activated by FcγRIIa) inhibitor	Fostamatinib	chronic ITP*	(127)
NADPH oxidase inhibitors	GSK2795039	HIT (E)	(52)
	DPI	HIT (E)	(52)
Autophagy			
Autophagy inducers			
mTOR inhibitor	Rapamycin (Sirolimus)	ITP (C)	(121)
AMPK/mTOR/ULK1 pathway inducer, autophagy gene expression, MEK/ERK pathway inducer	Vitamin D	ITP (C)	(128, 129)
Macroautophagy inducer	Retinoic acid	ITP (C)	(130)
Annexin A7 GTPase inhibitor	ABO	ITP (E)	(111)
Autophagy			
Autophagy inhibitors			
Lysosomal degradation inhibitors	Hydroxychloroquine	ITP (C)	(123, 128)
	Chloroquine	ITP (E)	(120)
PI3K inhibitor	3-MA	ITP (E)	(111)

ITP, immune thrombocytopenia; HIT, heparin-induced thrombocytopenia; iTTP, immune-mediated thrombotic thrombocytopenic purpura; NETs, neutrophil extracellular traps; DNase I, deoxyribonuclease I; PAD4, peptidylarginine deiminase 4; FcγRIIa, Fc gamma receptor II alpha; SYK, spleen tyrosine kinase; NADPH, nicotinamide adenine dinucleotide phosphate; AMPK, AMP-activated protein kinase; mTOR, mammalian target of rapamycin; ULK1, unc-51-like kinase 1; MEK, mitogen-activated protein kinase; ERK, extracellular-signal-regulated kinase; PI3K, phosphoinositide 3-kinase; ABO, 6-amino-2,3-dihydro-3-hydroxymethyl-1,4-benzoxazine; 3-MA, 3-methyladenine; DPI, diphenyleneiodonium chloride. Most of the currently available data are derived from experimental studies, whereas only a limited number of agents have been evaluated in clinical settings, primarily in ITP. For each target, representative drugs are listed together with their disease context, indicating whether the supporting evidence is derived from experimental models (E), clinical trials (C), or clinical use (*). Autophagy-modulating agents are further categorized as inducers or inhibitors, according to their mechanisms of action (e.g., mTOR inhibition, lysosomal blockade), and relevant references are provided for each entry.

In respect to autophagy treatment, *in vitro* studies indicate that modulation of autophagy with agents such as rapamycin or the autophagy inducer ABO directly affects platelet survival and function in ITP by enhancing autophagic activity and reducing platelet apoptosis via the PI3K-AKT-mTOR pathway (**Figure 2**) (111). Additional studies using autophagy inhibitors, such as 3-methyladenine (3-MA) in platelets and chloroquine in megakaryocytes, further implicate autophagy in the regulation of platelet production and maintenance (111, 120). Clinically, rapamycin has shown efficacy in refractory or relapsing ITP patients (121). Hydroxychloroquine was assessed in ITP associated with SLE or high titers of antinuclear antibodies and seemed to be safe and beneficial (122, 123). However, their evaluation in ITP, HIT, and iTTP remains limited, and, apart from fostamatinib in chronic ITP, none are currently in clinical use for these indications.

**Supplementary Table 1** identifies several drugs, which have not yet been investigated in the context of ITP, HIT, or iTTP, but may still be mechanistically relevant and emerge as potential candidates for off-label use in these diseases. Cytokine-directed therapies (e.g., TNF-α and IL-6 inhibitors), which are well established in diseases such as RA and Crohn’s disease and are under investigation in SLE and Sjögren’s syndrome, may hold promise in thrombo-inflammatory conditions. Agents targeting NET formation or enhancing NET clearance are the most attractive group potentially relevant in HIT and iTTP, and possibly in selected forms of ITP. A broad range of therapeutic strategies targeting NET formation and related inflammatory pathways has been explored in both experimental and clinical settings. These include MPO inhibitors (PF-1355, AZM198, PF-0628999, INV-315), NE inhibitors (sivelestat, POL6014, AZD9668, BAY85-8501, CHF6333), PAD4 inhibitors ((BB)-Cl-amidine/Cl-amidine, GSK484, JBI-589), and DNase-based approaches (DNase I, DNase1/DNase1L3). Collectively, these agents may limit NET-driven vascular and thrombotic injury where innate immune activation and immunothrombosis are key features. Complement-directed agents, such as eculizumab and avacopan, may also represent viable strategies, especially in iTTP, where complement activation may amplify endothelial injury.

Compounds modulating oxidative stress and NET-associated amplification pathways (e.g., N-acetylcysteine, MitoTempo, SKQ1, ethyl pyruvate, Metformin, and disulfiram) may further attenuate thrombo-inflammatory processes by reducing NET formation or inflammatory tissue damage.

Finally, autophagy modulators such as the mTOR inhibitor rapamycin, lysosomal inhibitors including hydroxychloroquine and chloroquine, and the CMA modulator P140 (Lupuzor), have demonstrated immunomodulatory effects in autoimmune diseases including SLE, RA, and APS. Among autophagy inducers, rapamycin, spermidine, resveratrol, and trichostatin A could theoretically restore immune homeostasis or reduce inflammatory injury. In contrast, autophagy inducers retinoic acid and vitamin D are more appropriately considered adjunctive supplements rather than primary therapeutic agents in this context. Autophagy inhibitors such as wortmannin, LY294002, Mdivi-1, P110, edaravone, SBI0206965, and GW406108X may be relevant because autophagy is linked to both immune activation and NET release. Notably, there remains a need for highly specific autophagy inhibitors. At present, however, therapeutic approaches targeting autophagy in autoimmune diseases remain largely exploratory.

Overall, NET inhibition, FcγRIIa/SYK signaling blockade, and complement modulation represent the most compelling unexploited therapeutic avenues, with the strongest conceptual rationale in HIT and iTTP. While autophagy-targeting strategies are mechanistically intriguing, their clinical relevance remains to be established.

A major limitation in this field is the lack of robust and physiologically relevant animal models that accurately reflect human thrombotic autoimmune diseases without artificial induction of pathology. This hampers the translation of experimental findings into clinical practice. Therefore, there is a clear unmet need for systematic investigation of inflammation-, NET-, and autophagy-targeting therapies in thrombotic autoimmune diseases, both in preclinical models and in well-designed clinical trials.

## 3 Conceptual hypothesis of the interplay of inflammation, autophagy, and NETs contributing to thrombosis and autoimmunity

The evidence discussed above supports a conceptual model in which inflammation, NET formation, and autophagy interact bidirectionally to sustain immunothrombosis in thrombotic autoimmune diseases (**Figure 1**). Inflammatory signaling promotes NET release, while NETs amplify vascular injury, coagulation, and autoantigen exposure. Autophagy, in turn, modulates both inflammatory responses and NET formation, positioning it as a central regulatory node in this network.

This interplay is supported by clinical observations. Pharmacological modulators of autophagy, such as rapamycin and chloroquine, are already used in autoimmune diseases including SLE and RA (**Supplementary Table 1**). Their efficacy highlights the relevance of autophagy-related pathways and suggests potential therapeutic implications for thrombotic autoimmune conditions such as ITP, HIT, and iTTP. However, the fact that both autophagy inducers and inhibitors can be beneficial underscores the context-dependent and complex role of autophagy in disease pathogenesis.

Within this framework, inflammation emerges as a key driver of tissue damage. Under physiological conditions, autophagy supports immune homeostasis by facilitating the clearance of damaged or dying cells and limiting excessive pro-inflammatory cytokine production (124, 125). When this process is impaired, cellular debris accumulates and inflammatory pathways, including LC3-associated phagocytosis, become overactivated, promoting sustained cytokine release and loss of immune tolerance (81).

NETs provide a direct mechanistic link between inflammation and thrombosis. By binding VWF and interacting with platelets and TF, they form a scaffold that promotes thrombus formation and coagulation (16, 17, 126). NET components such as DNA, histones, and HMGB1 further amplify inflammation, impair anticoagulant pathways, and inhibit fibrinolysis (17). In addition, they act as danger signals that activate immune cells and expose autoantigens, thereby reinforcing autoimmune responses.

Autophagy integrates these processes by regulating pathogen clearance, oxidative stress, and antigen presentation (1, 13, 81). Its dysregulation can therefore promote both inflammation and NET formation. Given its strong context dependence across cell types and inflammatory environments, the net effect of autophagy is likely to vary. Clarifying whether autophagic activity is insufficient or excessive in specific cellular compartments will be essential for understanding its role in thrombo-inflammatory autoimmune diseases.

## 4 Conclusion

Together, NET formation, inflammation, and autophagy form a dynamic and interconnected network that likely drives immunothrombosis in thrombotic autoimmune diseases. We propose a conceptual model of inflammation-driven NET formation and autophagy in thrombotic autoimmunity as depicted in **Figure 1**. Our central hypothesis proposes that autophagy acts as a modulatory mechanism that fine-tunes NET formation and inflammatory signaling in thrombotic autoimmune diseases. However, the precise molecular mechanisms underlying this interplay remain incompletely understood, particularly in ITP, HIT, and iTTP. Future studies dissecting cell type-specific autophagy pathways and their influence on NET-driven thrombosis may provide important insights and reveal new opportunities for therapeutic intervention aimed at restoring immune homeostasis to prevent thrombotic complications.
