# Supplementary material for: Autophagy, NET formation, and inflammation crosstalk in thrombotic autoimmune diseases
Source: Front Immunol. 2026 May 25;17:1844170. doi: 10.3389/fimmu.2026.1844170 (PMC13243073; doi:10.3389/fimmu.2026.1844170)
Supplement: Supplementary file 1 [file Table1.docx]

**Supplementary Table**

**Table S1**. This table summarizes therapeutic agents targeting key inflammatory pathways, NET formation, and autophagy processes alongside their corresponding level of evidences in autoimmune diseases including thrombotic autoimmune disorders ITP, HIT and iTTP and in other pathologies for which an association with thrombotic events has been reported. For each target, representative drugs are provided together with their associated disease context, specifying whether evidence derives from experimental models (E), clinical trials (C), or clinical use (*). Listed targets include cytokines (e.g., TNF-α, IL-6, type I interferons), intracellular signaling pathways (e.g., JAK, mTOR, AMPK), effector molecules involved in NET formation (e.g., MPO, NE, PAD4), and components of the complement and oxidative stress systems. Autophagy-modulating agents are further categorized as inducers or inhibitors, reflecting their mechanisms of action (e.g., mTOR inhibition, lysosomal blockade). References supporting each entry are provided.

| Target | Drug | Disease context | References |  |
| --- | --- | --- | --- | --- |
| Inflammation | | | |  |
| Intracellular receptor inhibitors | Glucocorticoids (Prednisone, Dexamethasone) | SLE*, RA*, ITP*, iTTP*, APS*, IBD* | (1-6) |  |
| TNF-α inhibitors | Infliximab | RA*, Crohn’s disease*, psoriasis*, psoriatic arthritis*, ankylosing spondylitis*, ulcerative colitis* | (7) |  |
|  | Adalimumab | RA*, Crohn’s disease*, psoriasis*, psoriatic arthritis*, ankylosing spondylitis*, noninfectious uveitis*, polyarticular JIA*, ulcerative colitis* | (7) |  |
|  | Etanecerpt | RA*, psoriasis*, psoriatic arthritis*, ankylosing spondylitis*, polyarticular JIA* | (7) |  |
| IL-6 receptor inhibitor | Tocilizumab | RA*, JIA*, giant cell arthritis*, ST-segment elevation myocardial infarction (C) | (8, 9) |  |
| IFN-α neutralization or INFRA1 inhibitors | Anifrolumab | SLE*, Sjögren’s syndrome (C) | (10, 11) |  |
|  | Sifalimumab | SLE* | (12) |  |
|  | Rontalizumab | SLE (C) | (13) |  |
|  | QX006N | SLE (C) | (14) |  |
| JAK inhibitor | Tofacitinib | SLE (C), RA*, psoriatic arthritis*, plaque psoriasis*, ulcerative colitis*, JIA* | (15-18) |  |
| Target | **Drug** | **Disease context** | **References** |  |
| NETs | | | |  |
| MPO inhibitor | | PF-1355 | Myocardial infarction (E), immune-complex vasculitis (E), anti-glomerular basement membrane glomerulonephritis (E) | (19, 20) |
|  | | Ceruloplasmin (plasma protein) | MPO plasma activity in mice (E) | (21) |
|  | | INV-315 | Coronary artery diseases (E), atherosclerosis (E) | (22, 23) |
|  | | PF-0628999 | Atherosclerosis (E) | (24) |
|  | | AZM198 | ANCA-associated vasculitis (E), obesity and hypertension (E), cresentic glomerulonephritis (E), atherosclerosis (E) | (25-28) |
| NE inhibitor | | Sivelestat | Severe acute pancreatitis (C), pulmonary fibrosis (E), COVID-19 (C), acute lung injury and acute respiratory distress syndrome* | (29-31) |
|  |  | POL6014 | Cystic fibrosis (C) | (32) |
|  |  | GW311616A | COPD (E) | (33) |
|  |  | CHF6333 | Bronchiectasis (C) | (34) |
|  |  | AZD9668 | Bronchiectasis (C), cystic fibrosis (C), COPD (C) | (35-37) |
|  |  | BAY85-8501 | Non-cystic fibrosis bronchiectasis (C) | (38) |
| DNA degradation | | DNase I | Cystic fibrosis*, COVID-19 (C), ischemia-reperfusion injury (E), RA (E), pathogen-induced lung injury (E), SLE (C), APS (E), IBD (E), iTTP (E), HIT (E) | (39-49) |
|  |  | DNase1/DNase1L3 | SLE (E) | (50) |
| PAD4 inhibitor | | (BB)-Cl-amidine or Cl-amidine | SLE (E), atherosclerosis (E), CIA (E), MPO-ANCA-associated vasculitis (E), IBD (E) | (46, 51-54) |
|  |  | GSK484 | Colorectal cancer (E), RA (E), healthy mouse and human neutrophils (E), colitis (E), HIT (E) | (49, 55-58) |
|  |  | JBI-589 | RA (E) | (59) |
| C5a inhibitor | | Eculizumab | Paroxysmal nocturnal hemoglobinuria*, atypical hemolytic uremic syndrome*, SLE (C) | (60-62) |
| C5a receptor inhibitor | | Avacopan | ANCA-associated vasculitis* | (63) |
| FcγRIIa inhibitors | | FcγRIIa antibody | VITT (E), HIT (E) | (49, 64-67) |
| SYK (activated by FcγRIIa) | | Fostamatinib | COVID-19 (E), chronic ITP*, RA (C) | (68-70) |
| ROS targeting agents | | NAC | SLE (C), ulcerative colitis (C), RA (C) | (71-73) |
|  |  | MitoTempo | SLE (E), IBD (E) | (74, 75) |
|  |  | SKQ1 | Chronic granulomatous disease (E), arthritis (E) | (76, 77) |
|  |  | Ethyl pyruvate | Sepsis (E) | (78) |
| PKC-NADPH oxidase pathway inhibitor | | Metformin | Type 2 diabetes (C) | (79) |
| NADPH oxidase inhibitors | | GSK2795039 | HIT (E) | (64) |
|  | | DPI | HIT (E) | (64) |
| Citrullinated histones H2A, H4 inhibitor | | tACPA | CAIA (E) | (80) |
|  |  | CIT-013 | RA (E), neutrophilic airway inflammation (E) | (81) |
| Citrullinated histone H3 inhibitor | | Anti-citrullinated H3 monoclonal antibody | Septic shock (E) | (82) |
| Casdermin D-mediated pore formation blocker | | Disulfiram | Transfusion-related acute lung injury (E), COVID-19 (E), sepsis (E), acute pancreatitis (E) | (83-85) |
| Pentraxin 3 inhibitor | | Atorvastatin | Inflammation (E) | (86) |
| Target | | **Drug** | **Disease context** | **References** |
| Autophagy | |  |  |  |
| Autophagy inducers | |  |  |  |
| mTOR inhibitor | | Rapamycin (Sirolimus) | RA (C), SLE (C), psoriasis (E), APS (C), ITP (C), EAE (E), Sjögren’s syndrome (E) | (87-94) |
|  |  | Everolimus | EAU (E), autoimmune hepatitis (C) | (95, 96) |
| AMPK/mTOR/ULK1 pathway inducer, autophagy gene expression, MEK/ERK pathway inducer | | Vitamin D | Psoriasis (C), MS (C), RA (E), SLE (C), type 1 diabetes (C), IBD*, ITP (C) | (97-100) |
| Macroautophagy inducer | | Retinoic acid | RA (E), SLE (C), IBD (E), MS (C), type 1 diabetes (E), ITP (C) | (101-103) |
| Macroautophagy inducer | | Spermidine (natural metabolite) | Psoriasis (E), SLE (E), type 1 diabetes (E), EAE (E) | (104-107) |
| AMPK pathway inducer, SIRT activator | | Resveratrol | EAE/MS (E), IBD (E), SLE (E), RA (C), systemic sclerosis (E), type 1 diabetes (E), pulmonary fibrosis (E), graves disease (E), psoriasis (E) | (108-110) |
| HDAC inhibitor - | | Trichostatin A | SLE (E), EAE (E), CIA (E) | (111, 112) |
| Annexin A7 GTPase inhibitor | | ABO | ITP (E) | (113) |
| Target | | **Drug** | **Disease context** | **References** |
| Autophagy | |  |  |  |
| Autophagy inhibitors | |  |  |  |
| Lysosomal degradation inhibitors | | Hydroxychloroquine | RA*, SLE*, Sjögren’s syndrome*, APS*, MS (C), ITP (C) | (99, 114-118) |
|  | | Chloroquine | RA*, SLE*, Sjögren’s syndrome*, APS*, ITP (E) | (114, 119) |
| HSPA8 inhibitor targeting CMA | | Lupuzor | SLE (C) | (120) |
| PI3K inhibitors | | 3-MA | RA (E), ITP (E), EAM (E), SLE (E) | (113, 121-123) |
|  |  | Wortmannin | PMA-induced NET formation (E) | (124, 125) |
|  |  | LY294002 | EAM (E) | (126) |
| Drp1 inhibitor | | Mdivi-1 | EAE (E), RA (E), type 1 diabetes (E) | (127-129) |
|  |  | P110 | EAE (E) | (130) |
| ROS scavenger | | Edaravone | Systemic sclerosis (E), autoimmune myocarditis (E), EAE (E), RA (E) | (131-134) |
| ULK1 inhibitors | | SBI0206965 | Psoriasis (E) | (135) |
|  | | GW406108X | SLE (E), RA (E) | (136) |

NETs, neutrophil extracellular traps; MPO, myeloperoxidase; NE, neutrophil elastase; DNase I, deoxyribonuclease I; PAD4, peptidylarginine deiminase 4; ROS, reactive oxygen species; RA, rheumatoid arthritis; SLE, systemic lupus erythematosus; ITP, immune thrombocytopenia; HIT, heparin-induced thrombocytopenia; VITT, vaccine-induced immune thrombotic thrombocytopenia; iTTP, immune-mediated thrombotic thrombocytopenic purpura; APS, antiphospholipid syndrome; MS, multiple sclerosis; IBD, inflammatory bowel disease; JIA, juvenile idiopathic arthritis; COVID-19, coronavirus disease 19; COPD, chronic obstructive pulmonary disease; EAE, experimental autoimmune encephalitis; EAU, experimental autoimmune uveoretinitis; EAM, experimental autoimmune myocarditis; CAIA, acute collagen antibody-induced arthritis; CIA, collagen-induced arthritis; tACPA, therapeutic anti-citrullinated protein antibody; SIRT, sirtuin; ABO, 6-amino-2,3-dihydro-3-hydroxymethyl-1,4-benzoxazine; TNF-α, tumor necrosis factor-α; IL-6, interleukin-6; IFN-α, interferon-α; IFNRA1, interferon alpha and beta receptor subunit 1; JAK, janus kinase; ANCA, anti-neutrophil cytoplasmic antibody; FcγRIIa, Fc gamma receptor IIa; C5a, complement component 5a; SYK, spleen tyrosine kinase; NAC, N-acetyl cysteine; SKQ1, 10-(6′-plastoquinonyl)decyltriphenylphosphonium; PKC, protein kinase C; NADPH**,** nicotinamide adenine dinucleotide phosphate; H2A, histone 2A; H3, histone 3; H4, histone 4; mTOR, mammalian target of rapamycin; AMPK, AMP-activated protein kinase; ULK1, unc-51-like kinase 1; MEK, mitogen-activated protein kinase kinase; ERK, extracellular-signal-regulated kinase; HSPA8, heat shock protein family A member 8; CMA, chaperone-mediated autophagy; PI3K, phosphoinositide 3-kinase; DPI, diphenyleneiodonium chloride; 3-MA, 3-methyladenine; PMA, phorbol 12-myristate 13-acetate; Drp1, dynamin-related protein 1; Mdivi-1, mitochondrial division inhibitor.

1. Martin-Iglesias D, Paredes-Ruiz D, Ruiz-Irastorza G. Use of Glucocorticoids in SLE: A Clinical Approach. *Mediterr J Rheumatol* (2024) 35:342-53. doi: 10.31138/mjr.230124.uos.

2. Prasad P, Verma S, Surbhi, Ganguly NK, Chaturvedi V, Mittal SA. Rheumatoid arthritis: advances in treatment strategies. *Mol Cell Biochem* (2023) 478:69-88. doi: 10.1007/s11010-022-04492-3.

3. Zufferey A, Kapur R, Semple JW. Pathogenesis and Therapeutic Mechanisms in  Immune Thrombocytopenia (ITP). *J Clin Med* (2017) 6:16. doi: 10.3390/jcm6020016.

4. Kremer Hovinga JA, Coppo P, Lämmle B, Moake JL, Miyata T, Vanhoorelbeke K. Thrombotic thrombocytopenic purpura. *Nat Rev Dis Primers* (2017) 3:17020. doi: 10.1038/nrdp.2017.20.

5. Zhu QN, Qi XB, Ren SW, Li YY, Yan ZW, Sun Y, et al. Novel advances on pathophysiological mechanisms, clinical manifestations, and treatment of antiphospholipid syndrome. *Front Immunol* (2025) 16:1639065. doi: 10.3389/fimmu.2025.1639065.

6. Bruscoli S, Febo M, Riccardi C, Migliorati G. Glucocorticoid Therapy in Inflammatory Bowel Disease: Mechanisms and Clinical Practice. *Front Immunol* (2021) 12:691480. doi: 10.3389/fimmu.2021.691480.

7. Jang DI, Lee AH, Shin HY, Song HR, Park JH, Kang TB, et al. The Role of Tumor Necrosis Factor Alpha (TNF-α) in Autoimmune Disease and Current TNF-α Inhibitors in Therapeutics. *Int J Mol Sci* (2021) 22:2719. doi: 10.3390/ijms22052719.

8. Choy EH, De Benedetti F, Takeuchi T, Hashizume M, John MR, Kishimoto T. Translating IL-6 biology into effective treatments. *Nat Rev Rheumatol* (2020) 16:335-45. doi: 10.1038/s41584-020-0419-z.

9. Kindberg KM, Broch K, Andersen G, Anstensrud AK, Åkra S, Woxholt S, et al. Neutrophil Extracellular Traps in ST-Segment Elevation Myocardial Infarction: Reduced by Tocilizumab and Associated With Infarct Size. *JACC Adv* (2024) 3:101193. doi: 10.1016/j.jacadv.2024.101193.

10. Kalunian KC, Furie R, Morand EF, Bruce IN, Manzi S, Tanaka Y, et al. A Randomized, Placebo-Controlled Phase III Extension Trial of the Long-Term Safety and Tolerability of Anifrolumab in Active Systemic Lupus Erythematosus. *Arthritis Rheumatol* (2023) 75:253-65. doi: 10.1002/art.42392.

11. Schiepek T, Grün P, Turhani F, Grün AS, Holzhauer S, Turhani D. Marked improvement of oral manifestations in systemic lupus erythematosus after therapy with IFNAR1 blocking antibody (anifrolumab): A case report. *Int J Surg Case Rep* (2025) 133:111593. doi: 10.1016/j.ijscr.2025.111593.

12. Khamashta M, Merrill JT, Werth VP, Furie R, Kalunian K, Illei GG, et al. Sifalimumab, an anti-interferon-α monoclonal antibody, in moderate to severe systemic lupus erythematosus: a randomised, double-blind, placebo-controlled study. *Ann Rheum Dis* (2016) 75:1909-16. doi: 10.1136/annrheumdis-2015-208562.

13. Kalunian KC, Merrill JT, Maciuca R, McBride JM, Townsend MJ, Wei X, et al. A Phase II study of the efficacy and safety of rontalizumab (rhuMAb interferon-α) in patients with systemic lupus erythematosus (ROSE). *Ann Rheum Dis* (2016) 75:196-202. doi: 10.1136/annrheumdis-2014-206090.

14. Chen X, Ke H, Li W, Yin L, Chen W, Chen T, et al. Structural basis for the recognition of IFNAR1 by the humanized therapeutic monoclonal antibody QX006N for the treatment of systemic lupus erythematosus. *Int J Biol Macromol* (2024) 268:131721. doi: 10.1016/j.ijbiomac.2024.131721.

15. Hasni SA, Gupta S, Davis M, Poncio E, Temesgen-Oyelakin Y, Carlucci PM, et al. Phase 1 double-blind randomized safety trial of the Janus kinase inhibitor tofacitinib in systemic lupus erythematosus. *Nat Commun* (2021) 12:3391. doi: 10.1038/s41467-021-23361-z.

16. Cohen SB, Tanaka Y, Mariette X, Curtis JR, Lee EB, Nash P, et al. Long-term safety of tofacitinib up to 9.5 years: a comprehensive integrated analysis of the rheumatoid arthritis clinical development programme. *RMD Open* (2020) 6:e001395. doi: 10.1136/rmdopen-2020-001395.

17. Zhao Z, Ye C, Dong L. The off-label uses profile of tofacitinib in systemic rheumatic diseases. *Int Immunopharmacol* (2020) 83:106480. doi: 10.1016/j.intimp.2020.106480.

18. Ruperto N, Brunner HI, Synoverska O, Ting TV, Mendoza CA, Spindler A, et al. Tofacitinib in juvenile idiopathic arthritis: a double-blind, placebo-controlled, withdrawal phase 3 randomised trial. *Lancet* (2021) 398:1984-96. doi: 10.1016/s0140-6736(21)01255-1.

19. Ali M, Pulli B, Courties G, Tricot B, Sebas M, Iwamoto Y, et al. Myeloperoxidase inhibition improves ventricular function and remodeling after experimental myocardial infarction. *JACC Basic Transl Sci* (2016) 1:633-43. doi: 10.1016/j.jacbts.2016.09.004.

20. Zheng W, Warner R, Ruggeri R, Su C, Cortes C, Skoura A, et al. PF-1355, a mechanism-based myeloperoxidase inhibitor, prevents immune complex vasculitis and anti-glomerular basement membrane glomerulonephritis. *J Pharmacol Exp Ther* (2015) 353:288-98. doi: 10.1124/jpet.114.221788.

21. Chapman AL, Mocatta TJ, Shiva S, Seidel A, Chen B, Khalilova I, et al. Ceruloplasmin is an endogenous inhibitor of myeloperoxidase. *J Biol Chem* (2013) 288:6465-77. doi: 10.1074/jbc.M112.418970.

22. Chaikijurajai T, Tang WHW. Myeloperoxidase: a potential therapeutic target for coronary artery disease. *Expert Opin Ther Targets* (2020) 24:695-705. doi: 10.1080/14728222.2020.1762177.

23. Liu C, Desikan R, Ying Z, Gushchina L, Kampfrath T, Deiuliis J, et al. Effects of a novel pharmacologic inhibitor of myeloperoxidase in a mouse atherosclerosis model. *PLoS One* (2012) 7:e50767. doi: 10.1371/journal.pone.0050767.

24. Roth Flach RJ, Su C, Bollinger E, Cortes C, Robertson AW, Opsahl AC, et al. Myeloperoxidase inhibition in mice alters atherosclerotic lesion composition. *PLoS One* (2019) 14:e0214150. doi: 10.1371/journal.pone.0214150.

25. Morris A, Geetha D. Advances in remission induction therapy for ANCA-associated vasculitis. *Best Pract Res Clin Rheumatol* (2023) 37:101828. doi: 10.1016/j.berh.2023.101828.

26. Piek A, Koonen DPY, Schouten EM, Lindtstedt EL, Michaëlsson E, de Boer RA, et al. Pharmacological myeloperoxidase (MPO) inhibition in an obese/hypertensive mouse model attenuates obesity and liver damage, but not cardiac remodeling. *Sci Rep* (2019) 9:18765. doi: 10.1038/s41598-019-55263-y.

27. Antonelou M, Michaëlsson E, Evans RDR, Wang CJ, Henderson SR, Walker LSK, et al. Therapeutic myeloperoxidase inhibition attenuates neutrophil activation, ANCA-mediated endothelial damage, and crescentic GN. *J Am Soc Nephrol* (2020) 31:350-64. doi: 10.1681/asn.2019060618.

28. Cheng D, Talib J, Stanley CP, Rashid I, Michaëlsson E, Lindstedt EL, et al. Inhibition of MPO (Myeloperoxidase) Attenuates Endothelial Dysfunction in Mouse Models of Vascular Inflammation and Atherosclerosis. *Arterioscler Thromb Vasc Biol* (2019) 39:1448-57. doi: 10.1161/atvbaha.119.312725.

29. Xie J, Lei R, Pei H, Gu Y, Zhang L, Liu J, et al. Effect and safety of sivelestat on acute severe pancreatitis with systemic inflammatory response syndrome: a retrospective study. *Sci Rep* (2025) 15:150. doi: 10.1038/s41598-024-84600-z.

30. Zeng W, Song Y, Wang R, He R, Wang T. Neutrophil elastase: From mechanisms to therapeutic potential. *J Pharm Anal* (2023) 13:355-66. doi: 10.1016/j.jpha.2022.12.003.

31. Kido T, Muramatsu K, Yatera K, Asakawa T, Otsubo H, Kubo T, et al. Efficacy of early sivelestat administration on acute lung injury and acute respiratory distress syndrome. *Respirology* (2017) 22:708-13. doi: 10.1111/resp.12969.

32. Barth P, Bruijnzeel P, Wach A, Sellier Kessler O, Hooftman L, Zimmermann J, et al. Single dose escalation studies with inhaled POL6014, a potent novel selective reversible inhibitor of human neutrophil elastase, in healthy volunteers and subjects with cystic fibrosis. *J Cyst Fibros* (2020) 19:299-304. doi: 10.1016/j.jcf.2019.08.020.

33. Wang K, Liao Y, Li X, Wang R, Zeng Z, Cheng M, et al. Inhibition of neutrophil elastase prevents cigarette smoke exposure-induced formation of neutrophil extracellular traps and improves lung function in a mouse model of chronic obstructive pulmonary disease. *Int Immunopharmacol* (2023) 114:109537. doi: 10.1016/j.intimp.2022.109537.

34. Gramegna A, Amati F, Terranova L, Sotgiu G, Tarsia P, Miglietta D, et al. Neutrophil elastase in bronchiectasis. *Respir Res* (2017) 18:211. doi: 10.1186/s12931-017-0691-x.

35. Stockley R, De Soyza A, Gunawardena K, Perrett J, Forsman-Semb K, Entwistle N, et al. Phase II study of a neutrophil elastase inhibitor (AZD9668) in patients with bronchiectasis. *Respir Med* (2013) 107:524-33. doi: 10.1016/j.rmed.2012.12.009.

36. Elborn JS, Perrett J, Forsman-Semb K, Marks-Konczalik J, Gunawardena K, Entwistle N. Efficacy, safety and effect on biomarkers of AZD9668 in cystic fibrosis. *Eur Respir J* (2012) 40:969-76. doi: 10.1183/09031936.00194611.

37. Vogelmeier C, Aquino TO, O'Brien CD, Perrett J, Gunawardena KA. A randomised, placebo-controlled, dose-finding study of AZD9668, an oral inhibitor of neutrophil elastase, in patients with chronic obstructive pulmonary disease treated with tiotropium. *Copd* (2012) 9:111-20. doi: 10.3109/15412555.2011.641803.

38. Watz H, Nagelschmitz J, Kirsten A, Pedersen F, van der Mey D, Schwers S, et al. Safety and efficacy of the human neutrophil elastase inhibitor BAY 85-8501 for the treatment of non-cystic fibrosis bronchiectasis: A randomized controlled trial. *Pulm Pharmacol Ther* (2019) 56:86-93. doi: 10.1016/j.pupt.2019.03.009.

39. Yang C, Montgomery M. Dornase alfa for cystic fibrosis. *Cochrane Database Syst Rev* (2021) 3:CD001127. doi: 10.1002/14651858.CD001127.pub5.

40. Porter JC, Inshaw J, Solis VJ, Denneny E, Evans R, Temkin MI, et al. Anti-inflammatory therapy with nebulized dornase alfa for severe COVID-19 pneumonia: a randomized unblinded trial. *Elife* (2024) 12:RP87030. doi: 10.7554/eLife.87030.

41. Wang S, Xie T, Sun S, Wang K, Liu B, Wu X, et al. DNase-1 treatment exerts protective effects in a rat model of intestinal ischemia-reperfusion injury. *Sci Rep* (2018) 8:17788. doi: 10.1038/s41598-018-36198-2.

42. Wang N, Ma J, Song W, Zhao C. An injectable hydrogel to disrupt neutrophil extracellular traps for treating rheumatoid arthritis. *Drug Deliv* (2023) 30:2173332. doi: 10.1080/10717544.2023.2173332.

43. Lefrançais E, Mallavia B, Zhuo H, Calfee CS, Looney MR. Maladaptive role of neutrophil extracellular traps in pathogen-induced lung injury. *JCI Insight* (2018) 3:e98178. doi: 10.1172/jci.insight.98178.

44. Davis JC, Jr., Manzi S, Yarboro C, Rairie J, McInnes I, Averthelyi D, et al. Recombinant human Dnase I (rhDNase) in patients with lupus nephritis. *Lupus* (1999) 8:68-76. doi: 10.1191/096120399678847380.

45. Meng H, Yalavarthi S, Kanthi Y, Mazza LF, Elfline MA, Luke CE, et al. In Vivo Role of Neutrophil Extracellular Traps in Antiphospholipid Antibody-Mediated Venous Thrombosis. *Arthritis Rheumatol* (2017) 69:655-67. doi: 10.1002/art.39938.

46. Drury B, Hardisty G, Gray RD, Ho GT. Neutrophil Extracellular Traps in Inflammatory Bowel Disease: Pathogenic Mechanisms and Clinical Translation. *Cell Mol Gastroenterol Hepatol* (2021) 12:321-33. doi: 10.1016/j.jcmgh.2021.03.002.

47. Yada N, Zhang Q, Bignotti A, Gralnek SH, Sosnovske D, Hogan K, et al. Targeting neutrophil extracellular trap accumulation under flow in patients with immune-mediated thrombotic thrombocytopenic purpura. *Blood Adv* (2024) 8:2536-51. doi: 10.1182/bloodadvances.2023011617.

48. Gollomp K, Kim M, Johnston I, Hayes V, Welsh J, Arepally GM, et al. Neutrophil accumulation and NET release contribute to thrombosis in HIT. *JCI Insight* (2018) 3:e99445. doi: 10.1172/jci.insight.99445.

49. Perdomo J, Leung HHL, Ahmadi Z, Yan F, Chong JJH, Passam FH, et al. Neutrophil activation and NETosis are the major drivers of thrombosis in heparin-induced thrombocytopenia. *Nat Commun* (2019) 10:1322. doi: 10.1038/s41467-019-09160-7.

50. Stabach PR, Sims D, Gomez-Bañuelos E, Zehentmeier S, Dammen-Brower K, Bernhisel A, et al. A dual-acting DNASE1/DNASE1L3 biologic prevents autoimmunity and death in genetic and induced lupus models. *JCI Insight* (2024) 9:e177003. doi: 10.1172/jci.insight.177003.

51. Knight JS, Subramanian V, O'Dell AA, Yalavarthi S, Zhao W, Smith CK, et al. Peptidylarginine deiminase inhibition disrupts NET formation and protects against kidney, skin and vascular disease in lupus-prone MRL/lpr mice. *Ann Rheum Dis* (2015) 74:2199-206. doi: 10.1136/annrheumdis-2014-205365.

52. Knight JS, Luo W, O'Dell AA, Yalavarthi S, Zhao W, Subramanian V, et al. Peptidylarginine deiminase inhibition reduces vascular damage and modulates innate immune responses in murine models of atherosclerosis. *Circ Res* (2014) 114:947-56. doi: 10.1161/circresaha.114.303312.

53. Willis VC, Gizinski AM, Banda NK, Causey CP, Knuckley B, Cordova KN, et al. N-α-benzoyl-N5-(2-chloro-1-iminoethyl)-L-ornithine amide, a protein arginine deiminase inhibitor, reduces the severity of murine collagen-induced arthritis. *J Immunol* (2011) 186:4396-404. doi: 10.4049/jimmunol.1001620.

54. Kusunoki Y, Nakazawa D, Shida H, Hattanda F, Miyoshi A, Masuda S, et al. Peptidylarginine Deiminase Inhibitor Suppresses Neutrophil Extracellular Trap Formation and MPO-ANCA Production. *Front Immunol* (2016) 7:227. doi: 10.3389/fimmu.2016.00227.

55. Wang B, Su X, Zhang B, Pan S. GSK484, an inhibitor of peptidyl arginine deiminase 4, increases the radiosensitivity of colorectal cancer and inhibits neutrophil extracellular traps. *J Gene Med* (2023) 25:e3530. doi: 10.1002/jgm.3530.

56. Ye H, Yang Q, Guo H, Wang X, Cheng L, Han B, et al. Internalisation of neutrophils extracellular traps by macrophages aggravate rheumatoid arthritis via Rab5a. *RMD Open* (2024) 10:e003847. doi: 10.1136/rmdopen-2023-003847.

57. Lewis HD, Liddle J, Coote JE, Atkinson SJ, Barker MD, Bax BD, et al. Inhibition of PAD4 activity is sufficient to disrupt mouse and human NET formation. *Nat Chem Biol* (2015) 11:189-91. doi: 10.1038/nchembio.1735.

58. Xie K, Hunter J, Lee A, Ahmad G, Witting PK, Ortiz-Cerda T. The PAD4 inhibitor GSK484 diminishes neutrophil extracellular trap in the colon mucosa but fails to improve inflammatory biomarkers in experimental colitis. *Biosci Rep* (2025) 45:375-97. doi: 10.1042/bsr20253205.

59. Gajendran C, Fukui S, Sadhu NM, Zainuddin M, Rajagopal S, Gosu R, et al. Alleviation of arthritis through prevention of neutrophil extracellular traps by an orally available inhibitor of protein arginine deiminase 4. *Sci Rep* (2023) 13:3189. doi: 10.1038/s41598-023-30246-2.

60. Kokoris S, Polyviou A, Evangelidis P, Grouzi E, Valsami S, Tragiannidis K, et al. Thrombosis in Paroxysmal Nocturnal Hemoglobinuria (PNH): From Pathogenesis to Treatment. *Int J Mol Sci* (2024) 25:12104. doi: 10.3390/ijms252212104.

61. Bryant A, Lecouturier J, Orozco-Leal G, Brocklebank V, Carnell S, Chadwick T, et al. Eculizumab withdrawal and monitoring in atypical haemolytic uraemic syndrome (SETS aHUS): a multicentre, open label, prospective, single arm trial. *Lancet Reg Health Eur* (2025) 56:101392. doi: 10.1016/j.lanepe.2025.101392.

62. Yamaguchi M, Mizuno M, Kitamura F, Iwagaitsu S, Nobata H, Kinashi H, et al. Case report: Thrombotic microangiopathy concomitant with macrophage activation syndrome in systemic lupus erythematosus refractory to conventional treatment successfully treated with eculizumab. *Front Med (Lausanne)* (2022) 9:1097528. doi: 10.3389/fmed.2022.1097528.

63. Jayne DRW, Merkel PA, Schall TJ, Bekker P. Avacopan for the treatment of ANCA-associated vasculitis. *N Engl J Med* (2021) 384:599-609. doi: 10.1056/NEJMoa2023386.

64. Leung HHL, Perdomo J, Ahmadi Z, Yan F, McKenzie SE, Chong BH. Inhibition of NADPH oxidase blocks NETosis and reduces thrombosis in heparin-induced thrombocytopenia. *Blood Adv* (2021) 5:5439-51. doi: 10.1182/bloodadvances.2020003093.

65. Leung HHL, Perdomo J, Ahmadi Z, Zheng SS, Rashid FN, Enjeti A, et al. NETosis and thrombosis in vaccine-induced immune thrombotic thrombocytopenia. *Nat Commun* (2022) 13:5206. doi: 10.1038/s41467-022-32946-1.

66. Greinacher A, Selleng K, Palankar R, Wesche J, Handtke S, Wolff M, et al. Insights in ChAdOx1 nCoV-19 vaccine-induced immune thrombotic thrombocytopenia. *Blood* (2021) 138:2256-68. doi: 10.1182/blood.2021013231.

67. Carnevale R, Leopizzi M, Dominici M, d'Amati G, Bartimoccia S, Nocella C, et al. PAD4-Induced NETosis Via Cathepsin G-Mediated Platelet-Neutrophil Interaction in ChAdOx1 Vaccine-Induced Thrombosis-Brief Report. *Arterioscler Thromb Vasc Biol* (2023) 43:e396-e403. doi: 10.1161/atvbaha.123.319522.

68. Strich JR, Ramos-Benitez MJ, Randazzo D, Stein SR, Babyak A, Davey RT, et al. Fostamatinib Inhibits Neutrophils Extracellular Traps Induced by COVID-19 Patient Plasma: A Potential Therapeutic. *J Infect Dis* (2021) 223:981-4. doi: 10.1093/infdis/jiaa789.

69. Bussel J, Arnold DM, Grossbard E, Mayer J, Treliński J, Homenda W, et al. Fostamatinib for the treatment of adult persistent and chronic immune thrombocytopenia: Results of two phase 3, randomized, placebo-controlled trials. *Am J Hematol* (2018) 93:921-30. doi: 10.1002/ajh.25125.

70. Genovese MC, Kavanaugh A, Weinblatt ME, Peterfy C, DiCarlo J, White ML, et al. An oral Syk kinase inhibitor in the treatment of rheumatoid arthritis: a three-month randomized, placebo-controlled, phase II study in patients with active rheumatoid arthritis that did not respond to biologic agents. *Arthritis Rheum* (2011) 63:337-45. doi: 10.1002/art.30114.

71. Lai ZW, Hanczko R, Bonilla E, Caza TN, Clair B, Bartos A, et al. N-acetylcysteine reduces disease activity by blocking mammalian target of rapamycin in T cells from systemic lupus erythematosus patients: a randomized, double-blind, placebo-controlled trial. *Arthritis Rheum* (2012) 64:2937-46. doi: 10.1002/art.34502.

72. Masnadi Shirazi K, Sotoudeh S, Masnadi Shirazi A, Moaddab SY, Nourpanah Z, Nikniaz Z. Effect of N-acetylcysteine on remission maintenance in patients with ulcerative colitis: A randomized, double-blind controlled clinical trial. *Clin Res Hepatol Gastroenterol* (2021) 45:101532. doi: 10.1016/j.clinre.2020.08.010.

73. Esalatmanesh K, Jamali A, Esalatmanesh R, Soleimani Z, Khabbazi A, Malek Mahdavi A. Effects of N-acetylcysteine supplementation on disease activity, oxidative stress, and inflammatory and metabolic parameters in rheumatoid arthritis patients: a randomized double-blind placebo-controlled trial. *Amino Acids* (2022) 54:433-40. doi: 10.1007/s00726-022-03134-8.

74. Lood C, Blanco LP, Purmalek MM, Carmona-Rivera C, De Ravin SS, Smith CK, et al. Neutrophil extracellular traps enriched in oxidized mitochondrial DNA are interferogenic and contribute to lupus-like disease. *Nat Med* (2016) 22:146-53. doi: 10.1038/nm.4027.

75. Wang A, Keita Å V, Phan V, McKay CM, Schoultz I, Lee J, et al. Targeting mitochondria-derived reactive oxygen species to reduce epithelial barrier dysfunction and colitis. *Am J Pathol* (2014) 184:2516-27. doi: 10.1016/j.ajpath.2014.05.019.

76. Vorobjeva N, Galkin I, Pletjushkina O, Golyshev S, Zinovkin R, Prikhodko A, et al. Mitochondrial permeability transition pore is involved in oxidative burst and NETosis of human neutrophils. *Biochim Biophys Acta Mol Basis Dis* (2020) 1866:165664. doi: 10.1016/j.bbadis.2020.165664.

77. Andreev-Andrievskiy AA, Kolosova NG, Stefanova NA, Lovat MV, Egorov MV, Manskikh VN, et al. Efficacy of Mitochondrial Antioxidant Plastoquinonyl-decyl-triphenylphosphonium Bromide (SkQ1) in the Rat Model of Autoimmune Arthritis. *Oxid Med Cell Longev* (2016) 2016:8703645. doi: 10.1155/2016/8703645.

78. Wang X, Sun S, Duan Z, Yang C, Chu C, Wang K, et al. Protective effect of ethyl pyruvate on gut barrier function through regulations of ROS-related NETs formation during sepsis. *Mol Immunol* (2021) 132:108-16. doi: 10.1016/j.molimm.2021.01.012.

79. Menegazzo L, Scattolini V, Cappellari R, Bonora BM, Albiero M, Bortolozzi M, et al. The antidiabetic drug metformin blunts NETosis in vitro and reduces circulating NETosis biomarkers in vivo. *Acta Diabetol* (2018) 55:593-601. doi: 10.1007/s00592-018-1129-8.

80. Chirivi RGS, van Rosmalen JWG, van der Linden M, Euler M, Schmets G, Bogatkevich G, et al. Therapeutic ACPA inhibits NET formation: a potential therapy for neutrophil-mediated inflammatory diseases. *Cell Mol Immunol* (2021) 18:1528-44. doi: 10.1038/s41423-020-0381-3.

81. van der Linden M, Kumari S, Montizaan D, van Dalen S, Kip A, Foster M, et al. Anti-citrullinated histone monoclonal antibody CIT-013, a dual action therapeutic for neutrophil extracellular trap-associated autoimmune diseases. *MAbs* (2023) 15:2281763. doi: 10.1080/19420862.2023.2281763.

82. Deng Q, Pan B, Alam HB, Liang Y, Wu Z, Liu B, et al. Citrullinated Histone H3 as a Therapeutic Target for Endotoxic Shock in Mice. *Front Immunol* (2019) 10:2957. doi: 10.3389/fimmu.2019.02957.

83. Adrover JM, Carrau L, Daßler-Plenker J, Bram Y, Chandar V, Houghton S, et al. Disulfiram inhibits neutrophil extracellular trap formation and protects rodents from acute lung injury and SARS-CoV-2 infection. *JCI Insight* (2022) 7:e157342. doi: 10.1172/jci.insight.157342.

84. Silva CMS, Wanderley CWS, Veras FP, Sonego F, Nascimento DC, Gonçalves AV, et al. Gasdermin D inhibition prevents multiple organ dysfunction during sepsis by blocking NET formation. *Blood* (2021) 138:2702-13. doi: 10.1182/blood.2021011525.

85. Han F, Chen H, Chen L, Yuan C, Shen Q, Lu G, et al. Inhibition of Gasdermin D blocks the formation of NETs and protects acute pancreatitis in mice. *Biochem Biophys Res Commun* (2023) 654:26-33. doi: 10.1016/j.bbrc.2023.02.082.

86. Baetta R, Lento S, Ghilardi S, Barbati E, Corsini A, Tremoli E, et al. Atorvastatin reduces long pentraxin 3 expression in vascular cells by inhibiting protein geranylgeranylation. *Vascul Pharmacol* (2015) 67-69:38-47. doi: 10.1016/j.vph.2014.11.008.

87. Lai ZW, Kelly R, Winans T, Marchena I, Shadakshari A, Yu J, et al. Sirolimus in patients with clinically active systemic lupus erythematosus resistant to, or intolerant of, conventional medications: a single-arm, open-label, phase 1/2 trial. *Lancet* (2018) 391:1186-96. doi: 10.1016/s0140-6736(18)30485-9.

88. Dufour I, Venot Q, Aydin S, Demoulin N, Canaud G, Morelle J. mTORC Pathway Activation and Effect of Sirolimus on Native Kidney Antiphospholipid Syndrome Nephropathy: A Case Report. *Am J Kidney Dis* (2020) 76:288-91. doi: 10.1053/j.ajkd.2019.08.032.

89. Kim HR, Kim JC, Kang SY, Kim HO, Park CW, Chung BY. Rapamycin Alleviates 2,3,7,8-Tetrachlorodibenzo-p-dioxin-Induced Aggravated Dermatitis in Mice with Imiquimod-Induced Psoriasis-Like Dermatitis by Inducing Autophagy. *Int J Mol Sci* (2021) 22:3968. doi: 10.3390/ijms22083968.

90. Feng Y, Meng H, Mu C, Zhang Y, Liu X, Shi Y, et al. Clinical study reveals the efficacy of sirolimus in treating primary immune thrombocytopenia: findings from a single-center study. *Blood Coagul Fibrinolysis* (2024) 35:155-60. doi: 10.1097/mbc.0000000000001303.

91. Byun S, Lee E, Lee KW. Therapeutic Implications of Autophagy Inducers in Immunological Disorders, Infection, and Cancer. *Int J Mol Sci* (2017) 18:1959. doi: 10.3390/ijms18091959.

92. Zhang F, Cheng T, Zhang SX. Mechanistic target of rapamycin (mTOR): a potential new therapeutic target for rheumatoid arthritis. *Arthritis Res Ther* (2023) 25:187. doi: 10.1186/s13075-023-03181-w.

93. Wang Y, Guo H, Liang Z, Feng M, Wu Y, Qin Y, et al. Sirolimus therapy restores the PD-1+ICOS+Tfh:CD45RA-Foxp3(high) activated Tfr cell balance in primary Sjögren's syndrome. *Mol Immunol* (2022) 147:90-100. doi: 10.1016/j.molimm.2022.04.006.

94. Hou H, Miao J, Cao R, Han M, Sun Y, Liu X, et al. Rapamycin Ameliorates Experimental Autoimmune Encephalomyelitis by Suppressing the mTOR-STAT3 Pathway. *Neurochem Res* (2017) 42:2831-40. doi: 10.1007/s11064-017-2296-7.

95. Hennig M, Bauer D, Wasmuth S, Busch M, Walscheid K, Thanos S, et al. Everolimus improves experimental autoimmune uveoretinitis. *Exp Eye Res* (2012) 105:43-52. doi: 10.1016/j.exer.2012.09.003.

96. Ytting H, Larsen FS. Everolimus treatment for patients with autoimmune hepatitis and poor response to standard therapy and drug alternatives in use. *Scand J Gastroenterol* (2015) 50:1025-31. doi: 10.3109/00365521.2014.998271.

97. Vincenzi F, Smirne C, Tonello S, Sainaghi PP. The Role of Vitamin D in Autoimmune Diseases. *Int J Mol Sci* (2026) 27:555. doi: 10.3390/ijms27010555.

98. Dell'Anna G, Fanizzi F, Zilli A, Furfaro F, Solitano V, Parigi TL, et al. The Role of Vitamin D in Inflammatory Bowel Diseases: From Deficiency to Targeted Therapeutics and Precise Nutrition Strategies. *Nutrients* (2025) 17:2167. doi: 10.3390/nu17132167.

99. Bockow B, Kaplan TB. Refractory immune thrombocytopenia successfully treated with high-dose vitamin D supplementation and hydroxychloroquine: two case reports. *J Med Case Rep* (2013) 7:91. doi: 10.1186/1752-1947-7-91.

100. Mabrouk RE, Hussein DT, Abbas M, Mabood SAE. Sufficient vitamin D is favorable for children with persistent and chronic immune thrombocytopenia. *Ann Hematol* (2023) 102:2033-8. doi: 10.1007/s00277-023-05210-9.

101. Zhang Y, Shi J, Xie Y, Shao H, Ning Y, Li Y. Exploring the supplementary potential of all-trans retinoic acid with methotrexate in rheumatoid arthritis: modulation of synovial cell apoptosis and autophagy. *Clin Exp Rheumatol* (2024) 42:1387-97. doi: 10.55563/clinexprheumatol/3pd9rp.

102. Zhang Y, Luo Y, Shi J, Xie Y, Shao H, Li Y. All-trans retinoic acid alleviates collagen-induced arthritis and promotes intestinal homeostasis. *Sci Rep* (2024) 14:1811. doi: 10.1038/s41598-024-52322-x.

103. Huang QS, Liu Y, Wang JB, Peng J, Hou M, Liu H, et al. All-trans retinoic acid plus high-dose dexamethasone as first-line treatment for patients with newly diagnosed immune thrombocytopenia: a multicentre, open-label, randomised, controlled, phase 2 trial. *Lancet Haematol* (2021) 8:e688-e99. doi: 10.1016/s2352-3026(21)00240-4.

104. Li G, Ding H, Yu X, Meng Y, Li J, Guo Q, et al. Spermidine Suppresses Inflammatory DC Function by Activating the FOXO3 Pathway and Counteracts Autoimmunity. *iScience* (2020) 23:100807. doi: 10.1016/j.isci.2019.100807.

105. Kim H, Massett MP. Effect of Spermidine on Endothelial Function in Systemic Lupus Erythematosus Mice. *Int J Mol Sci* (2024) 25:9920. doi: 10.3390/ijms25189920.

106. Karacay C, Prietl B, Harer C, Ehall B, Haudum CW, Bounab K, et al. The effect of spermidine on autoimmunity and beta cell function in NOD mice. *Sci Rep* (2022) 12:4502. doi: 10.1038/s41598-022-08168-2.

107. Yang Q, Zheng C, Cao J, Cao G, Shou P, Lin L, et al. Spermidine alleviates experimental autoimmune encephalomyelitis through inducing inhibitory macrophages. *Cell Death Differ* (2016) 23:1850-61. doi: 10.1038/cdd.2016.71.

108. Oliveira ALB, Monteiro VVS, Navegantes-Lima KC, Reis JF, Gomes RS, Rodrigues DVS, et al. Resveratrol Role in Autoimmune Disease-A Mini-Review. *Nutrients* (2017) 9:1306. doi: 10.3390/nu9121306.

109. Yu X, Chen M, Wu J, Song R. Research progress of SIRTs activator resveratrol and its derivatives in autoimmune diseases. *Front Immunol* (2024) 15:1390907. doi: 10.3389/fimmu.2024.1390907.

110. Wang D, Li SP, Fu JS, Zhang S, Bai L, Guo L. Resveratrol defends blood-brain barrier integrity in experimental autoimmune encephalomyelitis mice. *J Neurophysiol* (2016) 116:2173-9. doi: 10.1152/jn.00510.2016.

111. Reilly CM, Thomas M, Gogal R, Jr., Olgun S, Santo A, Sodhi R, et al. The histone deacetylase inhibitor trichostatin A upregulates regulatory T cells and modulates autoimmunity in NZB/W F1 mice. *J Autoimmun* (2008) 31:123-30. doi: 10.1016/j.jaut.2008.04.020.

112. Jayaraman A, Soni A, Prabhakar BS, Holterman M, Jayaraman S. The epigenetic drug Trichostatin A ameliorates experimental autoimmune encephalomyelitis via T cell tolerance induction and impaired influx of T cells into the spinal cord. *Neurobiol Dis* (2017) 108:1-12. doi: 10.1016/j.nbd.2017.07.015.

113. Wang CY, Ma S, Bi SJ, Su L, Huang SY, Miao JY, et al. Enhancing autophagy protects platelets in immune thrombocytopenia patients. *Ann Transl Med* (2019) 7:134. doi: 10.21037/atm.2019.03.04.

114. Schrezenmeier E, Dörner T. Mechanisms of action of hydroxychloroquine and chloroquine: implications for rheumatology. *Nat Rev Rheumatol* (2020) 16:155-66. doi: 10.1038/s41584-020-0372-x.

115. Li M, Weng L, Yu D, Yang G, Hao J. Increased formation of neutrophil extracellular traps induced by autophagy and identification of autophagy-related biomarkers in systemic lupus erythematosus. *Exp Dermatol* (2024) 33:e14881. doi: 10.1111/exd.14881.

116. Koch MW, Kaur S, Sage K, Kim J, Levesque-Roy M, Cerchiaro G, et al. Hydroxychloroquine for Primary Progressive Multiple Sclerosis. *Ann Neurol* (2021) 90:940-8. doi: 10.1002/ana.26239.

117. Blasco LM. Hydroxychloroquine alone for severe immune thrombocytopenic purpura associated with systemic lupus erythematosus. *Lupus* (2013) 22:752-3. doi: 10.1177/0961203313490239.

118. Khellaf M, Chabrol A, Mahevas M, Roudot-Thoraval F, Limal N, Languille L, et al. Hydroxychloroquine is a good second-line treatment for adults with immune thrombocytopenia and positive antinuclear antibodies. *Am J Hematol* (2014) 89:194-8. doi: 10.1002/ajh.23609.

119. Liu Z, Mei T. Immune thrombocytopenia induces autophagy and suppresses apoptosis in megakaryocytes. *Mol Med Rep* (2018) 18:4016-22. doi: 10.3892/mmr.2018.9373.

120. Zimmer R, Scherbarth HR, Rillo OL, Gomez-Reino JJ, Muller S. Lupuzor/P140 peptide in patients with systemic lupus erythematosus: a randomised, double-blind, placebo-controlled phase IIb clinical trial. *Ann Rheum Dis* (2013) 72:1830-5. doi: 10.1136/annrheumdis-2012-202460.

121. Feng C, Wang ZR, Li CY, Zhang XY, Wang XX. 3-MA attenuates collagen-induced arthritis in vivo via anti-inflammatory effect and autophagy inhibition. *BMC Musculoskelet Disord* (2025) 26:44. doi: 10.1186/s12891-025-08274-y.

122. Yuan J, Yu M, Li HH, Long Q, Liang W, Wen S, et al. Autophagy contributes to IL-17-induced plasma cell differentiation in experimental autoimmune myocarditis. *Int Immunopharmacol* (2014) 18:98-105. doi: 10.1016/j.intimp.2013.11.008.

123. Clarke AJ, Ellinghaus U, Cortini A, Stranks A, Simon AK, Botto M, et al. Autophagy is activated in systemic lupus erythematosus and required for plasmablast development. *Ann Rheum Dis* (2015) 74:912-20. doi: 10.1136/annrheumdis-2013-204343.

124. Remijsen Q, Vanden Berghe T, Wirawan E, Asselbergh B, Parthoens E, De Rycke R, et al. Neutrophil extracellular trap cell death requires both autophagy and superoxide generation. *Cell Res* (2011) 21:290-304. doi: 10.1038/cr.2010.150.

125. Itakura A, McCarty OJ. Pivotal role for the mTOR pathway in the formation of neutrophil extracellular traps via regulation of autophagy. *Am J Physiol Cell Physiol* (2013) 305:C348-54. doi: 10.1152/ajpcell.00108.2013.

126. Liu HS, Zhang J, Guo JL, Lin CY, Wang ZW. Phosphoinositide 3-kinase inhibitor LY294002 ameliorates the severity of myosin-induced myocarditis in mice. *Curr Res Transl Med* (2016) 64:21-7. doi: 10.1016/j.retram.2016.01.012.

127. Li YH, Xu F, Thome R, Guo MF, Sun ML, Song GB, et al. Mdivi-1, a mitochondrial fission inhibitor, modulates T helper cells and suppresses the development of experimental autoimmune encephalomyelitis. *J Neuroinflammation* (2019) 16:149. doi: 10.1186/s12974-019-1542-0.

128. Wang X, Chen Z, Fan X, Li W, Qu J, Dong C, et al. Inhibition of DNM1L and mitochondrial fission attenuates inflammatory response in fibroblast-like synoviocytes of rheumatoid arthritis. *J Cell Mol Med* (2020) 24:1516-28. doi: 10.1111/jcmm.14837.

129. Tang S, Huang M, Wang R, Li M, Dong N, Wu R, et al. Drp1-dependent mitochondrial fragmentation mediates photoreceptor abnormalities in type 1 diabetic retina. *Exp Eye Res* (2024) 242:109860. doi: 10.1016/j.exer.2024.109860.

130. Luo F, Herrup K, Qi X, Yang Y. Inhibition of Drp1 hyper-activation is protective in animal models of experimental multiple sclerosis. *Exp Neurol* (2017) 292:21-34. doi: 10.1016/j.expneurol.2017.02.015.

131. Yoshizaki A, Yanaba K, Ogawa A, Iwata Y, Ogawa F, Takenaka M, et al. The specific free radical scavenger edaravone suppresses fibrosis in the bleomycin-induced and tight skin mouse models of systemic sclerosis. *Arthritis Rheum* (2011) 63:3086-97. doi: 10.1002/art.30470.

132. Nimata M, Okabe TA, Hattori M, Yuan Z, Shioji K, Kishimoto C. MCI-186 (edaravone), a novel free radical scavenger, protects against acute autoimmune myocarditis in rats. *Am J Physiol Heart Circ Physiol* (2005) 289:H2514-8. doi: 10.1152/ajpheart.00661.2005.

133. Moriya M, Nakatsuji Y, Miyamoto K, Okuno T, Kinoshita M, Kumanogoh A, et al. Edaravone, a free radical scavenger, ameliorates experimental autoimmune encephalomyelitis. *Neurosci Lett* (2008) 440:323-6. doi: 10.1016/j.neulet.2008.05.110.

134. Arii K, Kumon Y, Ikeda Y, Suehiro T, Hashimoto K. Edaravone inhibits the disease activity in rheumatoid arthritis. *J Clin Pharm Ther* (2006) 31:197-9. doi: 10.1111/j.1365-2710.2006.00722.x.

135. Qiu X, Zheng L, Liu X, Hong D, He M, Tang Z, et al. ULK1 Inhibition as a Targeted Therapeutic Strategy for Psoriasis by Regulating Keratinocytes and Their Crosstalk With Neutrophils. *Front Immunol* (2021) 12:714274. doi: 10.3389/fimmu.2021.714274.

136. Nolan A, Foulkes DM, Fairweather EE, Alarcon MF, Linford C, Sellin A, et al. Autophagy inhibitors block pathogenic NET release in immune-mediated inflammatory disease without impairing host defence. *Rheumatology (Oxford)* (2025) 64:6409-14. doi: 10.1093/rheumatology/keaf437.
